# Classical and adaptive control of *ex vivo* skeletal muscle contractions using Functional Electrical Stimulation (FES)

**DOI:** 10.1371/journal.pone.0172761

**Published:** 2017-03-08

**Authors:** Paola Jaramillo Cienfuegos, Adam Shoemaker, Robert W. Grange, Nicole Abaid, Alexander Leonessa

**Affiliations:** 1 Department of Weapons and Systems Engineering, United States Naval Academy, Annapolis, Maryland, United States of America; 2 Department of Mechanical Engineering, Virginia Polytechnic Institute and State University, Blacksburg, Virginia, United States of America; 3 Department of Human Nutrition, Foods, and Exercise, Virginia Polytechnic Institute and State University, Blacksburg, Virginia, United States of America; 4 Department of Biomedical Engineering and Mechanics, Foods, Virginia Polytechnic Institute and State University, Blacksburg, Virginia, United States of America; Semmelweis Egyetem, HUNGARY

## Abstract

Functional Electrical Stimulation is a promising approach to treat patients by stimulating the peripheral nerves and their corresponding motor neurons using electrical current. This technique helps maintain muscle mass and promote blood flow in the absence of a functioning nervous system. The goal of this work is to control muscle contractions from FES via three different algorithms and assess the most appropriate controller providing effective stimulation of the muscle. An open-loop system and a closed-loop system with three types of model-free feedback controllers were assessed for tracking control of skeletal muscle contractions: a Proportional-Integral (PI) controller, a Model Reference Adaptive Control algorithm, and an Adaptive Augmented PI system. Furthermore, a mathematical model of a muscle-mass-spring system was implemented in simulation to test the open-loop case and closed-loop controllers. These simulations were carried out and then validated through experiments *ex vivo*. The experiments included muscle contractions following four distinct trajectories: a step, sine, ramp, and square wave. Overall, the closed-loop controllers followed the stimulation trajectories set for all the simulated and tested muscles. When comparing the experimental outcomes of each controller, we concluded that the Adaptive Augmented PI algorithm provided the best closed-loop performance for speed of convergence and disturbance rejection.

## Introduction

Functional Electrical Stimulation (FES) is a rehabilitation technique used to treat patients with absence of a functioning nervous system by maintaining blood flow and directly stimulating peripheral nerves [1, 2]. Motor unit recruitment varies with FES since fast motor units are recruited prior to slow motor units [2–4]. Although non-physiological recruitment attenuates muscle atrophy, it can induce fatigue which hinders muscle performance. Patterns of FES can be delivered via transcutaneous or percutaneous electrodes from an electrical stimulator which is regulated by a controller. The controller specifies stimulation parameters including amplitude, pulse width, and frequency. Theoretically, appropriate muscle contractions can be generated by FES to improve and maintain muscle function. To successfully activate the muscle, a balance must exist between the controlled level of contraction and the energy transfer from electrodes into the muscle. As demonstrated in [5–10], correlations exist between the number of tetanic contractions and muscle physical properties such as muscle mass, fiber size and force production. Assessment of muscle force and length changes during activation by electrical stimulation, which constitute reliable indicators of muscle function.

FES systems in clinical settings have relied primarily on open-loop control schemes. That is, there is no feedback captured from the stimulated muscle to compare to the desired muscle stimulus. Open-loop systems provide a command input but do not use output feedback to adjust the control action [11]. The major challenges when implementing closed-loop systems as rehabilitation tools include non-physiological recruitment due to synchronous recruitment, spasticity, complexity of muscle systems, appropriate sensing mechanisms, and disturbances to mention a few [12–14]. The designed control algorithm should be able to compensate and regulate muscles’ complexity as well as disturbances for optimal tracking.

Coordination of muscle activation is essential to movement. Cyclical activation by agonist-antagonistic muscle pairs provide motion at a joint (e.g., at the hip during walking), while synchronized contractions of the muscle pair stabilizes the joint [15]. A major motivation for this study was to develop a feedback control system to activate muscle. Popovic et al. [16] presents a review on various control methods used in simulation or experimentation, including open-loop controllers applying an inverse model, linear PID controllers, feedforward controllers with PID, and adaptive controllers. The increased need for effective and easy to operate FES equipment has motivated the exploration of these autonomous closed-loop systems, in which produced movement is measured in real-time using sensors and the stimulation pattern is modulated according to the difference between achieved and desired motion. For example, designed control systems for joint tracking by electrical stimulation includes a robust feedback system, H infinity controller, for ankle-moment tracking of the human calf muscles during upright position [17]; a neuro-PID controller for regulating knee angle employing an artificial neural network to regulate the desired knee angle to the appropriate stimulation parameters and a PID controller for compensation of unmodeled disturbances and tracking error [18]; and a sliding mode controller for regulating knee angle using Riener’s knee model [19]. The latter controller can display undesirable oscillatory behavior known as chattering [20, 21]. In addition, an example of adaptive control systems for muscle stimulation include a multiple-model adaptive control for upper-limb rehabilitation assuming nonlinear-time-invariant systems using time-varying Hammerstein structures to represent activation dynamics. This particular controller requires a set of subject models from experimental data and a set of tracking controllers from a healthy population [22].

Efficient controllers are needed to understand the relationships between the stimulation parameters and the resulting force and motion outputs. As a result, numerous issues arise regarding stability and robustness of controllers; for example, inefficient adjustments to fit the patient’s desired movement parameters, and sensitivities attributable to disturbances in the system make smooth coordinated movements difficult. Closed-loop systems enable repeatability through feedback by minimizing the error between the input and output of the system. In the present study, a closed-loop FES system was tested that compared different types of controllers to stimulate skeletal muscle *ex vivo*.

The key to implement an effective and efficient closed-loop FES system relies on four important criteria: (1) the system’s stability, which describes the boundedness of the input reference trajectory achieved by the muscle contraction, (2) the transient response, which provides a measure of how fast the muscle contractions converge to the reference trajectory, (3) the tracking performance, which scores the system’s ability to follow the desired motion, and (4) control constraints, which safeguard the muscles from unsuitable stimulation and joints from unwanted movements. Therefore, the controller design for a system comprised of a dynamic musculoskeletal model as well as sensors and actuators requires recurrent computation of the desired stimulation to accomplish the desired task [23–28]. In this study, we seek to test our controller detached from voluntary muscle contractions as suggested by Lynch and Popovic in [16]. Therefore, we dissected the muscle to asses the efficacy of the proposed control algorithms. This process guarantees the generated muscle contractions are solely due to the stimulation pulses set by the control effort.

The objective of this study was to test control of muscle contractions in simulation and experiments, specifically those of mouse muscle extensor digitorum longus (EDL) properties, via an open-loop system and a closed-loop system with three different closed-loop controllers to determine the most appropriate controller for position tracking. These controllers were based on minimizing the error of the system without relying on complex skeletal muscle models, such as proposed by the classic Hill muscle model [29], Huxley’s sliding filament muscle model [30], or rheological models [31]. We tested an open loop system and three model-free closed-loop controllers: Proportional-Integral (PI), Model Reference Adaptive Control (MRAC), and Adaptive Augmented PI (ADP-PI). Computational simulations were performed followed by validation through experiments *ex vivo*. Our goal was to determine which of these controllers provided the best performance of a skeletal muscle subjected to contraction when tracking each of four predetermined functions, including a step, sine, ramp, and square wave trajectories. Through adequate tuning of the parameters specified in each controller, we were able to minimize the error between the set desired trajectory and the actual muscle response.

## Materials and methods

The Materials and Methods section is divided into three main components: (1) the Skeletal Muscle Simulation, detailing the computational muscle system, (2) Controller Design, outlining the three controllers implemented in experiment and simulation, as well as (3) Experimental Design, which focuses on the explanation of the mouse muscle trials performed and the statistical analyses of these results. The nomenclature used throughout the paper is listed in the Supporting Information section, S1 Table.

### Skeletal muscle simulation

To assess the performance of open-loop and closed-loop controllers, a virtual setup comprised of a muscle-mass-spring system is modeled in MATLAB/Simulink. The muscle model algorithm and parameters used in simulation are adapted from the Thelen muscle model [32, 33], which is based on the Hill-type model [29] detailed in the Supporting Information sections, S1 Appendix and S2 Table. Four different trajectories (step, sine, ramp and square wave functions) were specified to carry out these simulations for the open-loop and closed-loop controllers.

**Fig 1:**
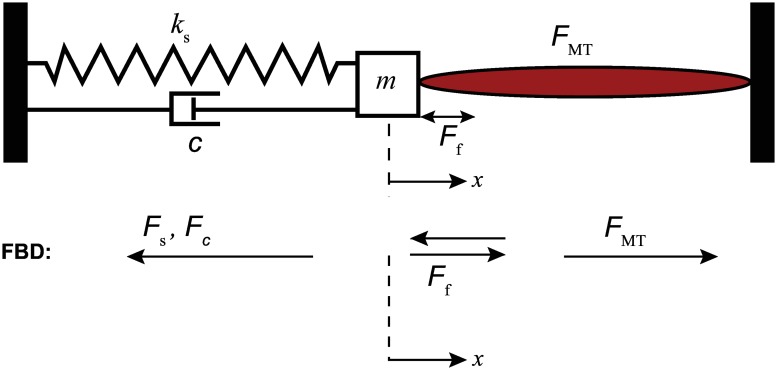
Muscle-mass-spring system schematic based on the system’s equation of motion. The EDL muscle contraction causes the mass positioned along the bar rail to move in the same direction as the contraction. The trajectory of the muscle is tracked using the linear magnetic encoder. The movement is then fed back to the controller.

The muscle-mass-spring system setup is shown in Fig 1. The muscle contraction *x* is recorded and serves as the feedback information for the closed-loop controllers. Dissipative forces such as Coulomb frictional force, *F*_f_, and damping force, $F_{c}=c\dot{x}$, are considered in the model since the experimental setup has the mass block moving along a steel rail. The static and kinetic coefficients of friction and the damping coefficient were chosen as *μ*_s_ = 0.120, *μ*_k_ = 0.080 and *c* = 3.500N-s/m, respectively. The spring constant *k*_s_ = 35.025N/m and mass *m* = 0.900g are based on the built experimental setup used to validate the simulations.

**Fig 2:**
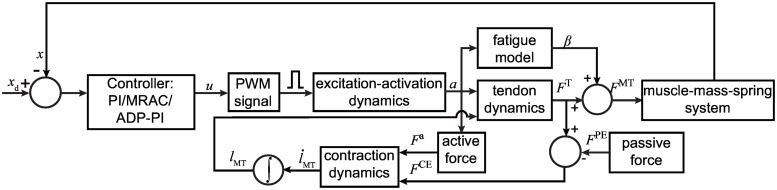
Flowchart showing the muscle-mass-spring system modeled in MATLAB/Simulink to test controllers.

A block diagram for the algorithm is shown in Fig 2. At the start of simulation, the muscle-tendon length, *l*_MT_, at *t* = 0, is set at maximum optimal fiber length $l_{0}^{M}$ and the tendon is set at the tendon slack length, $l_{s}^{T}$. The ratio between the EDL muscle fiber to whole muscle length, $l_{0}^{M}/l_{M}$, is 0.44 [34]. In a given time step, the muscle fiber length is calculated from the integration of the contraction dynamics block and updated into the tendon block. The force generated from the system, *F*^MT^, is the muscle force. A subsystem describes the motion dynamics of the system as follows,

$$\begin{array}{c} m\ddot{x}+c\dot{x}+k_{s}\left(x-x^{*}\right)=F^{\text{MT}}-F_{f}\text{sign}\;\left(\dot{x}\right), \end{array}$$ (1)

with initial conditions:

$$\begin{array}{cc} x(t=0)=0, & \dot{x}\left(t=0\right)=0. \end{array}$$ (2)

To implement *F*_f_, a switch is applied such that the system can vary between static and dynamic coefficients of friction, *μ*_s_ and *μ*_k_, respectively. The frictional force is determined by the following conditions:

$$\begin{array}{c} F_{f}=\left\{\begin{array}{cc} \mu_{k}mg\;\text{sign}\left(\dot{x}\right) & \text{if}\;|F^{\text{MT}}-c\dot{x}-k_{s}\left(x-x^{*}\right)|\;\geq \mu_{s}mg\\ \mu_{s}mg\;\text{sign}\left(\dot{x}\right) & \text{if}\;|F^{\text{MT}}-c\dot{x}-k_{s}\left(x-x^{*}\right)|\;<\mu_{s}mg \end{array}\right. \end{array}$$ (3)

The muscle contraction, *x*, was fedback to the system via the three closed-loop controllers. The controllers determined the appropriate activation time for the contraction pulse. For the open-loop case, tuning was done by inspection since there is no feedback in the system. A gain value was found to generate the pulse required for the muscle contraction to follow the specified trajectory.

### Controller design

For the muscle model simulation, an open-loop system and three closed-loop controllers were used to control muscle contraction. These were a linear PI control law and two nonlinear controllers which introduce adaptation to the system, MRAC and ADP-PI controllers. The following sections provide a tutorial explaining the structure of the three control techniques in detail. Furthermore, a brief sample outlining tuning of the controllers are detailed in the Supporting Information sections, S2 Appendix and S1 Fig.

#### Proportional integral controller

**Fig 3:**
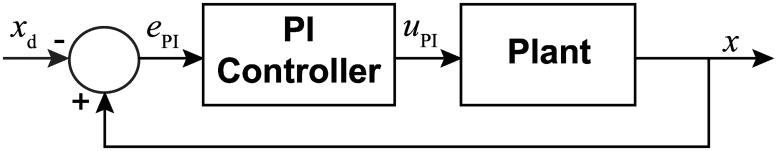
Block diagram of the closed-loop system using a Proportional Integral controller. For the PI control, the error *e*_PI_(*t*) is determined from the state *x*(*t*) and desired state *x*_d_(*t*). A control effort, *u*_PI_(*t*), based on *e*_PI_(*t*) is then input into the system.

For the purpose of muscle stimulation, a PI controller was used as described in [35, 36]. The structure of this controller is demonstrated in Fig 3. In this model, the user specifies the desired trajectory, *x*_d_(*t*), as the system input, which is then compared to the current measured state, *x*(*t*). Since a positive control force will result in negative contraction, the system error, *e*_PI_(*t*), is defined as follows,

$$\begin{array}{c} e_{\text{PI}}\left(t\right)≜x\left(t\right)-x_{d}\left(t\right). \end{array}$$ (4)

Based on this definition of the error, the PI controller then determines the control input, *u*_PI_(*t*), by implementing the following law,

$$\begin{array}{c} u_{\text{PI}}\left(t\right)=k_{P}\left(e_{\text{PI}}\left(t\right)+\frac{1}{T_{i}}\int_{0}^{t}e_{\text{PI}}\left(t\right)dt\right), \end{array}$$ (5)

where *k*_P_ is the proportional gain and *T*_i_ is the integral time. In standard form, the quantity $\frac{k_{P}}{T_{i}}$ is referred to as *k*_I_, commonly called the integral gain.

To tune the controller, a procedure was used similar to the Ziegler—Nichols Critical Gain method presented in [35]. With the integral gain set to *k*_I_ = 0, the proportional gain, *k*_P_, was steadily increased until oscillation was detected in a step input response. The optimal proportional gain was then taken to be the value used before oscillation was detected. Using this value, the reciprocal of the integral time, $\frac{1}{T_{i}}$, was increased until the step response error was determined to converge to a near zero value to ensure a proper transient response. Through appropriate tuning, the system may exhibit relatively small or no overshoot during the muscle contraction response.

#### Model Reference Adaptive Controller (MRAC)

**Fig 4:**
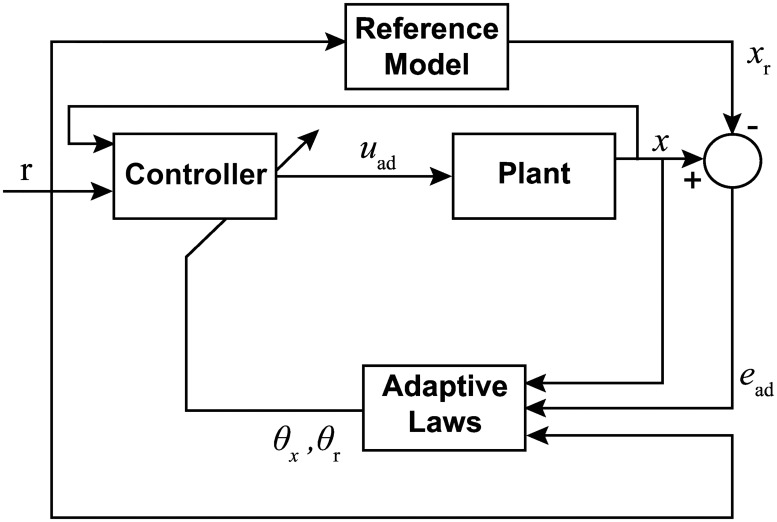
Block diagram of the closed loop system using a Model Reference Adaptive Controller. The error, *e*_ad_(*t*), state, *x*(*t*), and reference input, *r*(*t*), are used to define the dynamics for adaptive gains, *θ*_r_(*t*) and *θ*_*x*_(*t*). The control effort is computed using these gains, which then drives the mass-spring-muscle system to the reference system.

In addition to the PI controller, a MRAC algorithm based on the theory presented in [37] and [38] was used. In this design, seen in Fig 4, the control effort is a function of the adaptive gains, reference input, and current state. The adaptive gains, likewise, depend on the reference input, current state, and error between the unknown plant and reference system.

The following linear structure was implemented to describe the muscle behavior,

$$\begin{array}{c} \dot{x}\left(t\right)=ax\left(t\right)+bu_{\text{ad}}\left(t\right), \end{array}$$ (6)

where x(t)∈R is the system state, a∈R is an unknown system state constant, *b* > 0 is the system input constant with known sign and unknown magnitude, and $u_{\text{ad}}\left(t\right)\in R$ is the control effort. The MRAC algorithm will ultimately force the system given in Eq (6) to converge to the following reference model,

$$\begin{array}{c} {\dot{x}}_{r}\left(t\right)=a_{r}x_{r}\left(t\right)+b_{r}r\left(t\right), \end{array}$$ (7)

where $x_{r}\left(t\right)\in R$ is the reference model state, *a*_r_ < 0 is a negative (stable) system state constant, $b_{r}\in R$ is a known input constant, and r(t)∈R is the reference input. In the experiments, *a*_r_ is chosen such that *a*_r_ = −*b*_r_. The reference input, *r*(*t*), is then chosen so that the reference system *x*_r_(*t*) tracks the desired trajectory, $x_{d}\left(t\right)\in R$.

It is assumed that ideal gains, $\theta_{x}^{*}\in R$ and $\theta_{r}^{*}\in R$, exist to drive the system to the reference model through an ideal control law, $u_{\text{id}}\left(t\right)≜\theta_{x}^{*}x\left(t\right)+\theta_{r}^{*}r\left(t\right)$. By substituting *u*_id_(*t*) into Eq (6), the following relationship is obtained,

$$\begin{array}{c} \dot{x}\left(t\right)=\left(a+b\theta_{x}^{*}\right)x\left(t\right)+\left(b\theta_{r}^{*}\right)r\left(t\right). \end{array}$$ (8)

The error is given by $e_{\text{ad}}\left(t\right)≜x\left(t\right)-x_{r}\left(t\right)$. Choosing $\theta_{x}^{*}≜\frac{a_{r}-a}{b}$ and $\theta_{r}^{*}≜\frac{b_{r}}{b}$, the closed-loop system expression simplifies to the reference model and the error dynamics are given by

$$\begin{array}{c} {\dot{e}}_{\text{ad}}\left(t\right)=\dot{x}\left(t\right)-{\dot{x}}_{r}\left(t\right)=a_{r}x\left(t\right)-b_{r}r\left(t\right)-\left(a_{r}x_{r}\left(t\right)+b_{r}r\left(t\right)\right)=a_{r}e\left(t\right). \end{array}$$ (9)

Given that the reference model is chosen to be stable (*a*_r_ < 0), the error dynamics are also stable and the system state converges to the reference state. However, since *a* and *b* are unknown, the ideal gains $\theta_{x}^{*}$ and $\theta_{r}^{*}$ cannot be computed and the ideal control law *u*_id_(*t*) cannot be implemented. Hence, the ideal control law needs to be modified into an adaptive one which, instead of the ideal gains, implements adaptive gains, *θ*_*x*_(*t*) and *θ*_r_(*t*).

**Theorem 1**. Consider the system Eq (6), the reference system Eq (7) and adaptation laws given by

$$\begin{array}{c} {\dot{\theta }}_{x}\left(t\right)≜-\gamma_{x}e_{\text{ad}}\left(t\right)x\left(t\right)\text{sign}\left(b\right), \end{array}$$ (10)

$$\begin{array}{c} {\dot{\theta }}_{\text{r}}\left(t\right)≜-\gamma_{\text{r}}e_{\text{ad}}\left(t\right)r\left(t\right)\text{sign}\left(b\right), \end{array}$$ (11)

where *γ*_*x*_ > 0 and *γ*_r_ > 0 are the tuning parameters. Then, the closed loop system given by Eqs (6), (7), (10) and (11) with the adaptive control law

$$\begin{array}{c} u_{\text{ad}}\left(t\right)=\theta_{x}\left(t\right)x\left(t\right)+\theta_{r}\left(t\right)r\left(t\right), \end{array}$$ (12)

is Lyapunov stable and the tracking error *e*_ad_(*t*) converges to zero.

**Proof**. Define the difference between the adaptive and ideal gains as ${\tilde{\theta }}_{x}\left(t\right)≜\theta_{x}\left(t\right)-\theta_{x}^{*}$ and ${\tilde{\theta }}_{r}\left(t\right)≜\theta_{r}\left(t\right)-\theta_{r}^{*}$. By substituting the adaptive control input Eq (12) into Eq (6) and introducing the definitions of ${\tilde{\theta }}_{x}\left(t\right)$, ${\tilde{\theta }}_{r}\left(t\right)$, $\theta_{x}^{*}$, and $\theta_{r}^{*}$, we obtain,

$$\begin{array}{ccc} \dot{x}\left(t\right) & = & \left(a+b\theta_{x}\left(t\right)\right)x\left(t\right)+\left(b\theta_{r}\left(t\right)\right)r\left(t\right)\\ & = & \left(a+b\theta_{x}^{*}\right)x\left(t\right)+b\theta_{r}^{*}r\left(t\right)+b{\tilde{\theta }}_{x}\left(t\right)x\left(t\right)+b{\tilde{\theta }}_{r}\left(t\right)r\left(t\right)\\ & = & a_{r}x\left(t\right)+b_{r}r\left(t\right)+b{\tilde{\theta }}_{x}\left(t\right)x\left(t\right)+b{\tilde{\theta }}_{r}\left(t\right)r\left(t\right). \end{array}$$ (13)

Accordingly, the error dynamics are given by computing the difference between Eqs (13) and (7) as follows,

$$\begin{array}{c} {\dot{e}}_{\text{ad}}\left(t\right)≜a_{r}e_{\text{ad}}\left(t\right)+b{\tilde{\theta }}_{x}\left(t\right)x\left(t\right)+b{\tilde{\theta }}_{r}\left(t\right)r\left(t\right). \end{array}$$ (14)

Next, introduce the following Lyapunov function candidate [39],

$$\begin{array}{c} V\left(e_{\text{ad}}\left(t\right),{\tilde{\theta }}_{x}\left(t\right),{\tilde{\theta }}_{r}\left(t\right)\right)=\frac{1}{2}e_{\text{ad}}{\left(t\right)}^{2}+\frac{1}{2\gamma_{x}}\left|b\right|{\tilde{\theta }}_{x}{\left(t\right)}^{2}+\frac{1}{2\gamma_{r}}\left|b\right|{\tilde{\theta }}_{r}{\left(t\right)}^{2}. \end{array}$$ (15)

Using Eqs (14), (10) and (11), the Lyapunov derivative is determined by computing the time derivative of Eq (15) along the trajectories of Eq (14),

$$\begin{array}{ccc} \dot{V}\left(t\right) & ≜ & a_{r}e_{\text{ad}}\left(t\right){\dot{e}}_{\text{ad}}\left(t\right)+\frac{1}{\gamma_{x}}\left|b\right|{\tilde{\theta }}_{x}\left(t\right){\dot{\theta }}_{x}\left(t\right)+\frac{1}{\gamma_{r}}\left|b\right|{\tilde{\theta }}_{r}\left(t\right){\dot{\theta }}_{r}\left(t\right)\\ & = & a_{r}e_{\text{ad}}{\left(t\right)}^{2}\leq 0. \end{array}$$ (16)

Since *a*_r_ < 0, the LaSalle-Yoshizawa Theorem guarantees the error will converge to zero as time goes to infinity and all signals remain bounded [40].

Tuning the adaptive controller is a matter of adjusting the tuning constants *γ*_*x*_ and *γ*_r_ as well as the reference system parameters *a*_r_ and *b*_r_. While larger tuning constants will speed the rate of adaptation, high adaptive rates will lead to aggressive oscillatory behavior in the response, which is undesirable in muscle stimulation. On the other hand, limiting the rates of adaptation will also limit the rate of system convergence. Since the muscle stimulation occurred over a short period of time, added benefit was gained from adjusting the initial conditions of the adaptive gains, *θ*_*x*_(0) and *θ*_r_(0). This alteration allowed for the use of less aggressive adaptive rates by starting each experimental run with conditions closer to a converged state, but also required an additional tuning effort.

#### Adaptive augmented PI control

**Fig 5:**
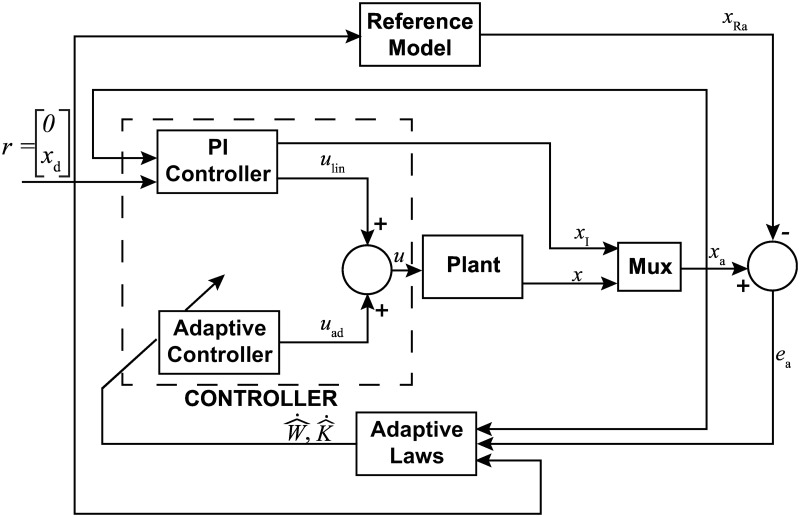
Block diagram of the closed-loop system using Adaptive augmented PI controller. The system assumes a single input-single output (SISO) form where *x*(*t*) is the state vector. To tune the controller, first we compute the linear gains, then calculate the adaptive parameters Γ_*K*_ and Γ_*W*_.

In order to maintain many of the overall benefits observed with the implementation of the PI controller but facilitate the advantages of adaptation, the ADP-PI controller presented in [41] was chosen. Alterations, however, were made to this strategy to support a more heuristic tuning methodology which was used with the classical PI controller. The design of this controller is given in Fig 5.

At the core of the algorithm, a classical PI controller provides a linear contribution to the overall control strategy, augmented with an adaptive control effort which depends on adaptive gains, current system state, desired trajectory, and a set of structured nonlinear functions of the state. The adaptive gains are continuously updated based on system state, trajectory, error between the reference system, and the same set of nonlinear functions. Using a first order model to describe the muscle behavior similar to that of the MRAC algorithm, the system model is given as follows,

$$\begin{array}{c} \dot{x}\left(t\right)=ax\left(t\right)+b\lambda^{*}\left(u\left(t\right)+W^{*T}\phi \left(x(t)\right)\right), \end{array}$$ (17)

where a∈R is a known state constant, b∈R is a known input constant, λ* > 0 is a constant of known sign and unknown magnitude, u(t)∈R is the control effort, $W^{*}\in R^{p}$ is an unknown constant vector where *p* > 0 is the number of linear and nonlinear functions contained in $\phi (x(t))\in R^{p}$. Note that there is no loss of generality in assuming the state constant *a* is known as the vector of basis functions *ϕ*(*x*(*t*)) will also contain the simple linear function *x*(*t*) hence providing an overall uncertainty over the linear coefficient of the system dynamics.

Since the PI control relies on the integration of the error between current and desired states, an additional integral state is introduced, $x_{I}\left(t\right)≜\int_{0}^{t}(x\left(\tau \right)-x_{d}\left(\tau \right))d\tau$. This state is then augmented to the current model to provide an overall representation,

$$\begin{array}{ccc} {\dot{x}}_{a}\left(t\right) & ≜ & \left[\begin{array}{cc} a & 0\\ 1 & 0 \end{array}\right]x_{a}\left(t\right)+\left[\begin{array}{c} b\\ 0 \end{array}\right]\lambda^{*}\left(u\left(t\right)+W^{*T}\phi \left(x(t)\right)\right)+\left[\begin{array}{c} 0\\ -1 \end{array}\right]x_{d}\left(t\right), \end{array}$$ (18)

where $x_{a}\left(t\right)≜{\left[x\left(t\right)\;x_{I}\left(t\right)\right]}^{T}$.

To implement the adaptive control algorithm, a reference system needs to be introduced. In particular, we chose the known dynamics of Eq (18) which can be written as

$$\begin{array}{c} {\dot{x}}_{\text{lin}}\left(t\right)=\left[\begin{array}{cc} a & 0\\ 1 & 0 \end{array}\right]x_{\text{lin}}\left(t\right)+\left[\begin{array}{c} b\\ 0 \end{array}\right]u_{\text{lin}}\left(t\right)+\left[\begin{array}{c} 0\\ -1 \end{array}\right]x_{d}\left(t\right), \end{array}$$ (19)

where $x_{\text{lin}}(t)\in R^{2}$. A standard PI controller is chosen to guarantee that Eq (19) tracks the desired trajectory, that is

$$\begin{array}{c} u_{\text{lin}}\left(t\right)≜-K^{*T}\left(x_{\text{lin}}\left(t\right)-\left[\begin{array}{c} x_{d}\left(t\right)\\ 0 \end{array}\right]\right), \end{array}$$ (20)

where $K^{*}≜{\left[k_{P}\;k_{I}\right]}^{T}$.

Substituting Eq (20) into Eq (19) provides the following closed-loop system, which will be used as the reference system for the design of the adaptive controller

$$\begin{array}{c} {\dot{x}}_{\text{lin}}\left(t\right)=A_{r}x_{\text{lin}}\left(t\right)+\left[\begin{array}{c} bk_{P}\\ -1 \end{array}\right]x_{d}\left(t\right), \end{array}$$ (21)

where *A*_r_ is defined as,

$$\begin{array}{c} A_{r}≜\left(\left[\begin{array}{cc} a & 0\\ 1 & 0 \end{array}\right]-\left[\begin{array}{c} b\\ 0 \end{array}\right]K^{*T}\right). \end{array}$$ (22)

By designing *k*_P_ and *k*_I_ through classical techniques, *A*_r_ is guaranteed to be Hurwitz. This, however, is under the assumption that both *a* and *b* are known. In reality, however, *k*_P_ and *k*_I_ are tuned in the same manner discussed with the PI controller. Once satisfactory values are determined, approximate values for *a* and *b* are found by examining the step response to achieve a similar trend in simulation.

To design a controller which guarantees that the augmented system Eq (18) converges to the reference system Eq (21), we introduce the following tracking error,

$$\begin{array}{c} e_{a}\left(t\right)≜x_{a}\left(t\right)-x_{\text{lin}}\left(t\right), \end{array}$$ (23)

and analyze its dynamics, obtained as follows,

$$\begin{array}{ccc} {\dot{e}}_{a}\left(t\right) & = & {\dot{x}}_{a}\left(t\right)-{\dot{x}}_{\text{lin}}\left(t\right)\\ & = & A_{r}e_{a}\left(t\right)+\left[\begin{array}{c} b\\ 0 \end{array}\right]\lambda^{*}\left(u\left(t\right)+W^{*T}\phi \left(x(t)\right)\right)+\left[\begin{array}{c} b\\ 0 \end{array}\right]K^{*T}x_{a}\left(t\right)+\left[\begin{array}{c} -bk_{P}\\ 0 \end{array}\right]x_{d}\left(t\right)\\ & = & A_{r}e_{a}\left(t\right)+\left[\begin{array}{c} b\\ 0 \end{array}\right]\lambda^{*}\left(u\left(t\right)+\frac{1}{\lambda^{*}}K^{*T}\left(x_{a}\left(t\right)-\left[\begin{array}{c} x_{d}\left(t\right)\\ 0 \end{array}\right]\right)+W^{*T}\phi \left(x(t)\right)\right). \end{array}$$ (24)

Next, we define the control effort *u*(*t*) as the sum of a linear part and an adaptive one, that is $u\left(t\right)≜u_{\text{lin}}\left(t\right)+u_{\text{ad}}\left(t\right)$, where *u*_lin_(*t*) is given by Eq (20) and *u*_ad_(*t*) needs to be designed. Accordingly, Eq (24) can be simplified to the following expression,

$$\begin{array}{ccc} {\dot{e}}_{a}\left(t\right) & = & A_{r}e_{a}\left(t\right)+\\ & & +\left[\begin{array}{c} b\\ 0 \end{array}\right]\lambda^{*}\left(u_{\text{ad}}\left(t\right)+W^{*T}\phi \left(x(t)\right)+\frac{1-\lambda^{*}}{\lambda^{*}}K^{*T}\left(x_{a}\left(t\right)-\left[\begin{array}{c} x_{d}\left(t\right)\\ 0 \end{array}\right]\right)\right). \end{array}$$ (25)

Since *A*_r_ is designed to be Hurwitz, by choosing *u*_ad_(*t*) as follows

$$\begin{array}{c} u_{\text{ad}}^{*}\left(t\right)≜-W^{*T}\phi \left(x(t)\right)-\frac{1-\lambda^{*}}{\lambda^{*}}K^{*T}\left(x_{a}\left(t\right)-\left[\begin{array}{c} x_{d}\left(t\right)\\ 0 \end{array}\right]\right), \end{array}$$ (26)

the error dynamics Eq (25) simplifies to ${\dot{e}}_{a}\left(t\right)=A_{r}e_{a}\left(t\right)$ which guarantees asymptotic stability and hence convergence of *e*_a_(*t*) to 0.

However, since the values of W* and λ* are unknown, the ideal controller Eq (26) cannot be implemented. Hence, the ideal control law needs to be modified into an adaptive one which, instead of those ideal values, implements adaptive variables $\hat{W}\left(t\right)$ and $\hat{K}\left(t\right)$.

**Theorem 2**. Consider the system Eq (18), the reference system Eq (21), and the adaptation laws given by

$$\begin{array}{c} \dot{\hat{K}}\left(t\right)=\text{sign}\left(\lambda^{*}\right)\Gamma_{K}\left(x_{\text{a}}\left(t\right)-\left[\begin{array}{c} x_{\text{d}}\left(t\right)\\ 0 \end{array}\right]\right)e_{\text{a}}^{\text{T}}\left(t\right)P\left[\begin{array}{c} b\\ 0 \end{array}\right], \end{array}$$ (27)

$$\begin{array}{c} \dot{\hat{W}}\left(t\right)=\text{sign}\left(\lambda^{*}\right)\Gamma_{W}\phi \left(x\left(t\right)\right)e_{\text{a}}^{\text{T}}\left(t\right)P\left[\begin{array}{c} b\\ 0 \end{array}\right], \end{array}$$ (28)

where Γ_*K*_ > 0 and Γ_*W*_ > 0 are the adaptation gains. The closed loop system given by Eqs (18), (21), (27) and (28) with the adaptive control law

$$\begin{array}{c} u_{\text{ad}}\left(t\right)≜-{\hat{W}}^{T}\left(t\right)\phi \left(x(t)\right)-{\hat{K}}^{T}\left(t\right)\left(x_{a}\left(t\right)-\left[\begin{array}{c} x_{d}\left(t\right)\\ 0 \end{array}\right]\right), \end{array}$$ (29)

is Lyapunov stable and the tracking error *e*_ad_(*t*) converges to zero.

**Proof**. By defining the difference between the ideal gains and the adaptive gains as $\tilde{K}\left(t\right)≜\hat{K}\left(t\right)-\frac{1-\lambda^{*}}{\lambda^{*}}K^{*}$ and $\tilde{W}\left(t\right)≜\hat{W}\left(t\right)-W^{*}$, the error dynamics can be rewritten as follows,

$$\begin{array}{ccc} {\dot{e}}_{a}\left(t\right) & = & A_{r}e_{a}\left(t\right)-\left[\begin{array}{c} b\\ 0 \end{array}\right]\lambda^{*}\left({\tilde{W}}^{T}\left(t\right)\phi \left(x(t)\right)+{\tilde{K}}^{T}\left(t\right)\left(x_{a}\left(t\right)-\left[\begin{array}{c} x_{d}\left(t\right)\\ 0 \end{array}\right]\right)\right). \end{array}$$ (30)

Next, the following Lyapunov function candidate is introduced,

$$\begin{array}{ccc} V(e_{a}\left(t\right),\tilde{K}\left(t\right),\tilde{W}\left(t\right)) & = & e_{a}^{T}\left(t\right)Pe_{a}\left(t\right)+\left|\lambda^{*}\right|{\tilde{K}}^{T}\left(t\right)\Gamma_{K}^{-1}\tilde{K}\left(t\right)+\left|\lambda^{*}\right|{\tilde{W}}^{T}\left(t\right)\Gamma_{W}^{-1}\tilde{W}\left(t\right), \end{array}$$ (31)

where $P\in R^{2\times 2}$ is a positive definite matrix. Using Eqs (30), (27) and (28), the Lyapunov derivative is determined by computing the time derivative of Eq (31) along the trajectories of Eq (30)

$$\begin{array}{ccc} \dot{V}\left(t\right) & = & {\dot{e}}_{a}^{T}\left(t\right)Pe_{a}\left(t\right)+e_{a}^{T}\left(t\right)P{\dot{e}}_{a}\left(t\right)+2\left|\lambda^{*}\right|{\tilde{K}}^{T}\left(t\right)\Gamma_{K}^{-1}\dot{\tilde{K}}\left(t\right)+2\left|\lambda^{*}\right|{\tilde{W}}^{T}\left(t\right)\Gamma_{W}^{-1}\dot{\tilde{W}}\left(t\right)\\ & = & e_{a}^{T}\left(t\right)\left(A_{r}^{T}P+PA_{r}\right)e_{a}\left(t\right)\\ & & +2\left|\lambda^{*}\right|{\tilde{K}}^{T}\left(t\right)\left(-\text{sign}\left(\lambda^{*}\right)+\left(x_{a}\left(t\right)-\left[\begin{array}{c} x_{d}\left(t\right)\\ 0 \end{array}\right]\right)e_{a}^{T}\left(t\right)P\left[\begin{array}{c} b\\ 0 \end{array}\right]+\Gamma_{K}^{-1}{\dot{\tilde{K}}}^{T}\left(t\right)\right)\\ & & +2\left|\lambda^{*}\right|{\tilde{W}}^{T}\left(t\right)\left(-\text{sign}\left(\lambda^{*}\right)\phi \left(x(t)\right)e_{a}^{T}\left(t\right)P\left[\begin{array}{c} b\\ 0 \end{array}\right]+\Gamma_{W}^{-1}{\dot{\tilde{W}}}^{T}\left(t\right)\right).\\ & = & e_{a}^{T}\left(t\right)\left(A_{r}^{T}P+PA_{r}\right)e_{a}\left(t\right)=-e_{a}^{T}\left(t\right)Qe_{a}\left(t\right)\leq 0, \end{array}$$ (32)

where the last inequality follows from the fact that $Q\in R^{2\times 2}$ is positive definite since it satisfies the Lyapunov algebraic equation $A_{r}^{T}P+PA_{r}+Q=0$ with *P* positive definite and *A*_r_ Hurwitz. Hence, LaSalle-Yoshizawa Theorem guarantees that *e*_a_(*t*) will converge to zero as time goes to infinity and all signals stay bounded [40].

### Experimental design

To validate the results obtained from simulation, experiments were carried out for the closed-loop controllers: PI, MRAC and ADP-PI systems. Since open-loop systems performances are not guaranteed to remain within acceptable limits, while closed-loop systems are known to be more accurate due to feedback, no open-loop systems were considered in experimentation. Thus, the main focus of the paper is the application of an optimal approach, i.e. closed-loop control, for muscle stimulation. In our experiments, the fast contracting EDL mouse muscle was activated at 100Hz to evoke tetanic contractions.

Twenty-four male C57/BL10 ScN mice with ages ranging from 12 to 32 weeks (Jackson Laboratories) were fed standard, irradiated Envigo Teklad Global 18% Protein Rodent Diet (2918) (Cambridgeshire, UK) and provided water *ad libitum*. They were maintained in a temperature controlled vivarium between 68°F–79°F on a 12 hour light/ dark cycle. Cages were maintained by vivarium staff as needed. Mean body mass was 28.04 ± 4.41 g. Mice were euthanized with CO_2_ to carry out the experiments. Eight mice were used (N = 8) for each type of controller. One muscle was used per animal. All procedures were approved by the Institutional Animal Care and Use Committee (IACUC No. 08-119-HNFE) at Virginia Tech. The approval was obtained prior to the start of the study. All efforts were made to minimize animal suffering. After euthanasia with CO_2_, one EDL muscle was surgically removed. Once removed, the muscle was incubated in a physiological salt solution gassed with 95%O_2_, 5%CO_2_ at room temperature (20°C). Since isolated muscle viability *ex vivo* decreases with increased temperature, we selected room temperature to maximize muscle viability [42]. The muscle aligned equidistant between two platinum electrodes. Temperature was maintained between 16°C–17°C. The EDL muscle was set at 1g of resting tension which corresponds to $l_{0}^{M}$, prior to the initial stimulation.

During muscle activation, mass trajectory was measured using a linear encoder (Renishaw LM15, IL). Feedback was captured from the instantaneous position to minimize the error, *e*(*t*), between the desired predefined trajectory and the actual muscle contraction. Each contraction trial was executed at a stimulation frequency of 100Hz with a duration ranging from 7s to 10s. The constant voltage applied from the stimulator (Aurora Scientific Inc., 701B stimulator) to the EDL muscle bath was set at 25V and a variable current limited to 1A. The resting tension at $l_{0}^{M}$ was sustained between each contraction; the muscle shortened 20% to 40% of $l_{0}^{M}$ during contractions [42]. Each EDL muscle was stimulated using either the PI, MRAC, or ADP-PI controller. Measurements were recorded, which primarily included muscle force, muscle contraction, and twitch responses. The latter were recorded at the beginning and between trials to monitor muscle fatigue and assess muscle decay. Furthermore, resting periods of one minute were provided between trials to ensure the quality of the muscle. The muscles’ predefined trajectories included step, sinusoidal, ramp, and square inputs. After the conclusion of each trial, physical measurements of the muscle including muscle mass and muscle length at resting tension were recorded. The average muscle mass and muscle length were 0.0142±0.0024g and 10.44±0.69mm, respectively. A timeline of events describing the step by step process for an experiment is detailed on Supporting Information S3 Table.

#### Controller setup

**Fig 6:**
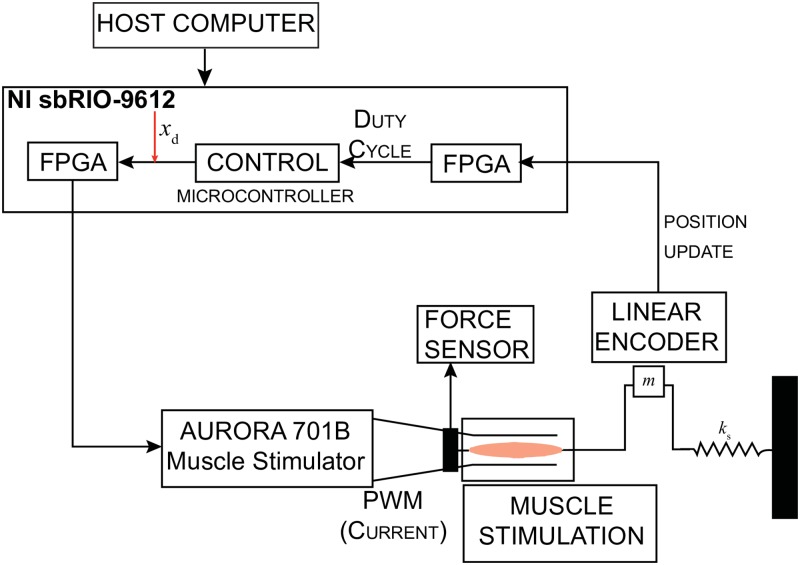
Controller setup displaying the interface between the controller and experimental setup. The control effort, *u*(*t*), determines the parameters of the duty cycle transmitted to the FPGA, which onsets the muscle contraction.

A host computer supported the exchange communication between the muscle’s experimental and controller setup, as displayed in Fig 6. The host program configured the data acquisition board NI sbRIO-9612 (National Instruments, TX) to update the parameters on the real-time controllers. The board recorded and sampled at 250Hz. The NI sbRIO-9612 operated a 400MHz microprocessor and a 2M field programmable gate array (FPGA). The FPGA outputted a digital pulse width modulation (PWM) signal at a frequency of 100 Hz, which consists of a succession of pulses in which the pulse duration is determined by the feedback controllers to output the adequate stimulation. The PWM signal set by the control effort, *u*(*t*), has a pulse duration saturation limit between 0*μ*s and a configurable upper limit of 200*μ*s. The FPGA sends the PWM stimulation signal via the digital output ports to the external trigger on the current stimulator, which are connected to the platinum electrodes.

Muscle contraction was measured through a magnetic linear encoder (Renishaw, IL) with a resolution of 5*μ*m. The signal from the encoder, comprised of two square waves in quadrature, was detected by the FPGA through the digital input pins at a rate of 1MHz. The high sample rate ensures all encoder pulses are considered. The position information from the FPGA was sampled at a rate of 250Hz. Simultaneously, the controller, running on the microprocessor, updated the output and pulse width parameters from the FPGA using the measured position. Moreover, position measurements, adaptive gains for the MRAC and the ADP-PI controller as well as desired trajectories were updated and saved on the host computer.

#### Statistical analyses

Four controllers (one open-loop and three closed-loop) were evaluated in simulation and the closed-loop controllers were validated experimentally. Four trajectories were implemented per controller. In the case of the simulations, tuned trajectories were attained (deterministic results). For the experimental results, trials were carried out for all trajectories. Mean and standard deviation (*x*_*μ*_ ± *σ*_*x*_) were determined for the squared tracking error (STE) between the desired and/or reference trajectory and the actual trajectory. STE is determined as,

$$\begin{array}{c} \text{STE}=\left\{\begin{array}{cc} \frac{1}{n}\sum_{i=1}^{n}{\left(x_{d}\left(t\right)-x\left(t\right)\right)}^{2}, & \left(\text{for PI controller}\right)\\ \frac{1}{n}\sum_{i=1}^{n}{\left(x_{r}\left(t\right)-x\left(t\right)\right)}^{2}, & (\text{for MRAC and ADP—PI controllers}). \end{array}\right. \end{array}$$

In addition, mean settling time $\left({\bar{t}}_{s}\right)$ for the step trajectory was calculated across all experiments per controller. To test significance, we evaluated normality visually through the quantile-quantile plot (Q-Q plot) of residuals and equality of variances via Levene’s test. To ensure normality, a square root transformation for the ramp and square trajectories was required. Furthermore, a Welch’s unequal variances t-test was performed. Since the data did not meet the homogeneity of variances assumption, we implemented the Games Howell post hoc test. Statistical analyses were performed using JMP (SAS Institute, Cary, North Carolina, USA). Significance was set at *α* < 0.05. The statistical analyses focused on identifying differences between all controllers. The number of samples for each trajectory are detailed on Table 1. We hypothesize the ADP-PI controller has the best performance overall.

**Table 1:** Number of trials from experiments used for statistical analysis.

Trajectory	PI	MRAC	ADP-PI
Step	33	52	20
Sine	14	15	21
Ramp	6	13	11
Square	11	9	14
N_total_	64	89	66

## Results

### Overall observations regarding tuning of the controller gains for the PI, MRAC, and ADP-PI

**Fig 7:**
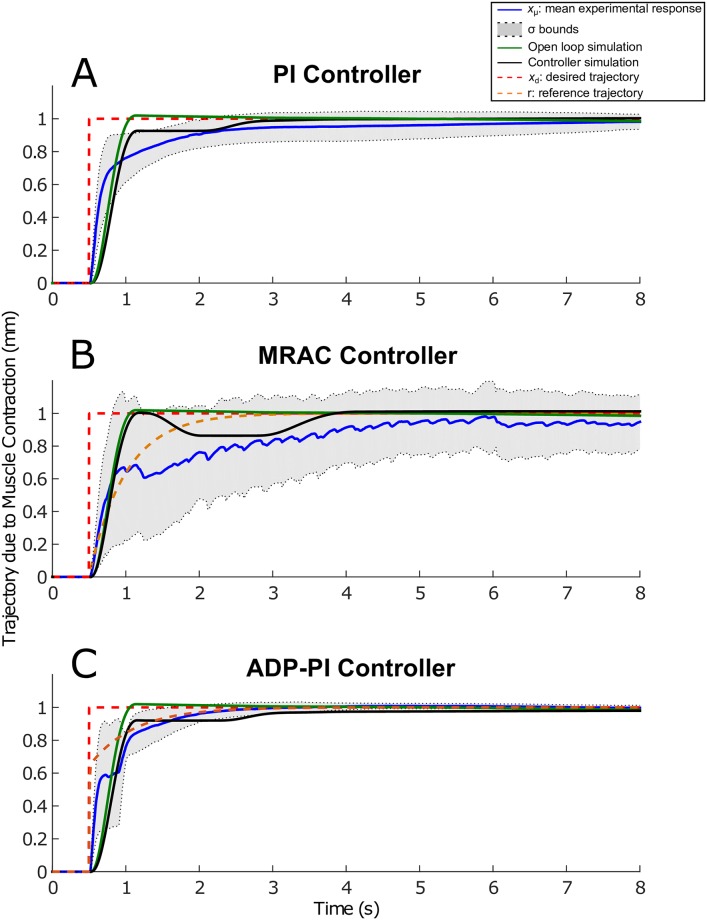
Step trajectories due to EDL muscle contraction using the PI, MRAC, and ADP-PI controllers from simulations and experiments. *x*_*μ*_ ± *σ* from experimental results as well as simulations, including the controllers and open-loop simulations, are shown to assess the controller performance in simulation and validate the outcome through experiments for the (A) PI controller, (B) MRAC controller, and (C) ADP-PI controller.

**Fig 8:**
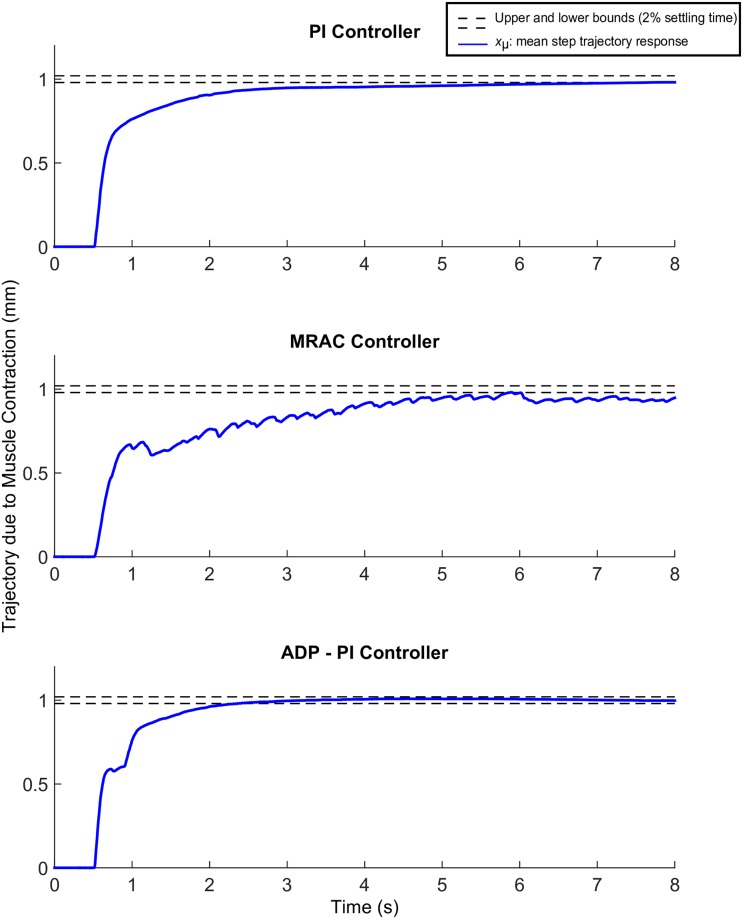
Mean experimental step trajectories due to EDL muscle contraction using the PI, MRAC, and ADP-PI controllers. The dashed lines represent the 2% upper and lower bounds to reach the desired trajectory set at 1mm.

**Fig 9:**
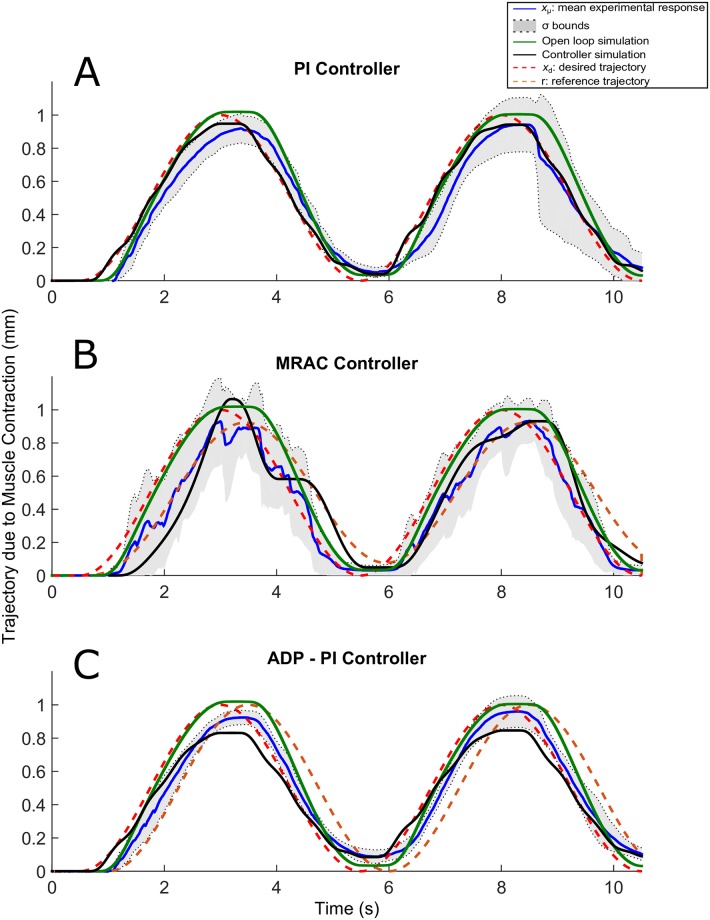
Sine trajectories due to EDL muscle contraction using the PI, MRAC, and ADP-PI controllers from simulations and experiments. *x*_*μ*_ ± *σ* from experimental results as well as simulations, including the controllers and open-loop simulations, are shown to assess the controller performance in simulation and validate the outcome through experiments for the (A) PI controller, (B) MRAC controller, and (C) ADP-PI controller.

**Fig 10:**
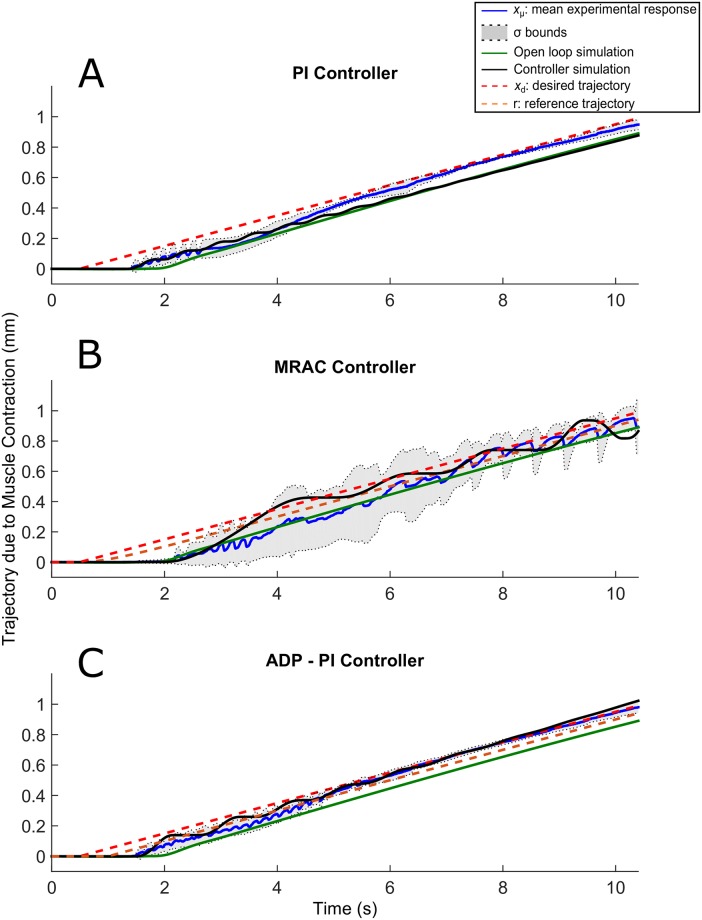
Ramp trajectories due to EDL muscle contraction using the PI, MRAC, and ADP-PI controllers from simulations and experiments. *x*_*μ*_ ± *σ* from experimental results as well as simulations, including the controllers and open-loop simulations, are shown to assess the controller performance in simulation and validate the outcome through experiments for the (A) PI controller, (B) MRAC controller, and (C) ADP-PI controller.

**Fig 11:**
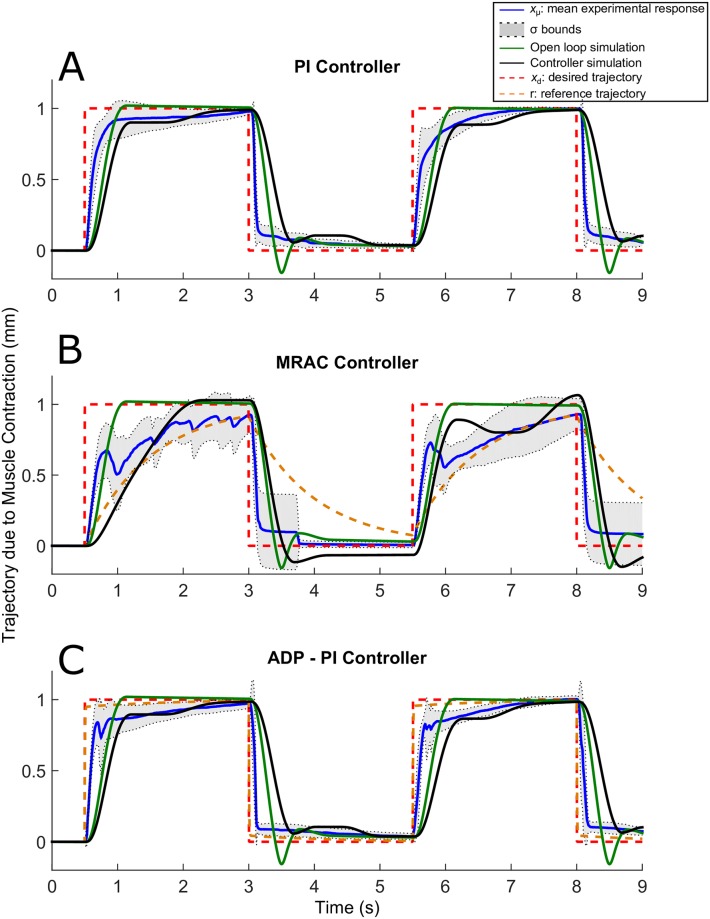
Square trajectories due to EDL muscle contraction using the PI, MRAC, and ADP-PI controllers from simulations and experiments. *x*_*μ*_ ± *σ* from experimental results as well as simulations, including the controllers and open-loop simulations, are shown to assess the controller performance in simulation and validate the outcome through experiments for the (A) PI controller, (B) MRAC controller, and (C) ADP-PI controller.

Tuning of parameter gains were largely dependent on each muscle. To determine the appropriate gains for the control effort, we applied first a step input for tuning the muscle gains. All the trajectory responses exhibited friction, which was mainly attributed to the mass motion along the rail. Results are displayed in Figs 7–11.

The PI controller gains, *k*_P_ and *k*_I_, are user-defined parameters which drive the response of the system. The input control effort obtained through the PI controller generated the appropriate PWM signal for muscle contraction. Examples of the pulse duration input signal and its corresponding output are shown in S2–S3 Figs in the Supporting Information.

The MRAC controller required six tuning parameters. The MRAC controlled trajectories presented fluctuations during adaptation of the controller’s parameters. A sample of these parameters are listed for simulation and experiments on Table 2. The adaptive gains, *θ*_*x*_(*t*) and *θ*_r_(*t*), are specified for *t* = 0, the tuning parameters, *γ*_*x*_ and *γ*_r_ ensures that *e*_ad_(*t*) converges to zero, the constants, *a*_r_ and *b*_r_ describe the muscle model behavior. Specifying these tuning parameters allowed the MRAC controller to determine the required control input signal generating the pulse duration for muscle contraction. The pulse duration and its corresponding trajectory response are shown in Supporting Information S4 and S5 Figs.

**Table 2:** MRAC adaptive parameters implemented for all trajectories for a trial experiment and simulation.

	*γ*_*x*_ × 10^5^ [*m*^−3^]	*γ*_r_ × 10^5^ [*m*^−3^]	*θ*_*x*_(0) [*s*/*m*]	*θ*_r_(0) [*s*/*m*]	*a*_r_ [1/*s*], -*b*_r_ [*m*/*s*^2^]
Step exp	1.00	1.00	0.025	0.040	−2.0
Step sim	1.45	1.45	0.035	0.140	−2.0
Sine exp	8.00	5.00	0.020	0.060	−2.0
Sine sim	9.50	2.50	0.010	0.060	−2.0
Ramp exp	8.00	5.00	0.020	0.060	−2.0
Ramp sim	10.50	8.50	0.080	0.060	−2.0
Square exp	8.00	5.00	0.020	0.060	−2.0
Square sim	1.00	1.00	0.025	0.040	−2.0

The ADP-PI controller was implemented assuming linear dynamics for muscle behavior. This nonlinear controller, which defines the control input, *u*(*t*), is composed of a linear and adaptive component. For the ADP-PI controller, the function *ϕ*(*x*(*t*)) was chosen as,

$$\begin{array}{c} \phi \left(x(t)\right)=\left[x^{2},e^{x},\sin\left(x\right),x\right], \end{array}$$ (33)

which was arbitrarily selected based on the muscle controlled trajectories. The known state constant *a* and the known input constant *b* were determined through computational simulations using previously calculated PI gains, *k*_P_ and *k*_I_. These were then used to determine the *P* matrix by solving $A_{r}^{T}P+PA_{r}+Q=0$, prior to the start of each trial.

The ADP-PI controller converged at a rate proportional to muscle fiber contraction, thus minimizing the error between the reference trajectory and the actual trajectory. This was observed throughout all trajectories using the ADP-PI controller. The controller required tuning of ten parameters, which are listed in Table 3, for the simulation and experimental validation. First, the linear gains, *k*_P_ and *k*_I_ were computed using a method similar to the Ziegler—Nichols Critical Gain method presented in [35]. The constants, *a* and *b* are parameters determined for the linear muscle model behavior as described by Eq (17). The adaptive gains, Γ_*K*_ and Γ_*W*_, in Table 3 are initial values set at *t* = 0. In this way, the system can converge quickly through adaptation. Moreover, S6 and S7 Figs Design in the Supporting Information displayed the pulse duration input signal and its corresponding output for all trajectories in experiment. Trajectory dispersion was smaller compared to the PI and MRAC results as determined by the *σ* bounds.

**Table 3:** Parameters of the ADP-PI controller including the linear gains, *k*_P_ and *k*_I_, and state constants: *a*, *b* to describe the first order model for muscle behavior.

	*k*_P_ [*s*/*m*]	*k*_I_ [1/*m*]	*a* [1/*s*]	*b* [*m*/*s*^2^]	Γ_*K*_1__, Γ_*K*_2__ × 10^−1^ [*s*^2^/*m*^3^]	Γ_*W*_1__, Γ_*W*_2__, Γ_*W*_3__, Γ_*W*_4__ × 10^−4^ [*s*^2^/*m*^2^]
Step exp	0.050	0.75	-11.55	2.89	0.10	0.10
Step sim	0.090	0.40	-3.50	8.50	0.10	0.10
Sine exp	0.055	1.00	-11.55	2.89	1.00	1.00
Sine sim	0.400	0.25	-1.50	12.50	1.00	1.00
Ramp exp	0.035	0.90	-57.75	2.89	1.00	1.00
Ram sim	0.080	0.90	-3.50	8.50	1.00	1.00
Square exp	0.055	1.00	-50.0	2.89	1.00	1.00
Square sim	0.075	0.37	-3.50	10.50	1.00	1.00

### Muscle contractions following step input trajectories show faster convergence using the ADP-PI controller

The results obtained implementing the step input trajectory for the three controllers are displayed in Fig 7A–7C. The plots include mean experimental step trajectories (*x*_*μ*_ ± *σ*), controller simulations, and open-loop simulations as well as the desired and reference step input trajectories. Overall, no overshoot was observed for all the controllers. The mean and standard deviation (*x*_*μ*_ ± *σ* bounds) displayed faster convergence to the reference trajectory using ADP-PI controller when compared to the PI and MRAC controllers. Moreover, trajectory dispersion was smaller using the ADP-PI controller when compared to the PI and MRAC results as determined by the *σ* bounds.

We evaluated the mean settling times for convergence using the PI, MRAC and ADP-PI controllers as shown in Fig 8, that is, the time response to reach and maintain 2% of the final value, set at 1mm. The mean settling time using the PI controller was ${\bar{t}}_{\text{s}}=6.5\text{s}$, while the mean settling time using the MRAC controller exhibited fluctuations and did not settle during the 8s of muscle stimulation. The mean settling time using the ADP-PI controller is reached at ${\bar{t}}_{s}=2.3\text{s}$, which is almost three times faster than using the PI controller.

Comparing the mean settling times to simulations for each controller, we observed the PI controller simulation settling at *t*_s_ = 3.4s while remaining within the variance bounds of the experimental results (refer to Fig 7A) and in turn capturing the muscle activation behavior similar to the experiments, while the open-loop simulation converged much faster at *t*_s_ = 1.1s. The MRAC controller simulation (as shown in Fig 7B) converged after 3.8s to the reference trajectory. The open-loop trajectory responded rapidly to the reference trajectory and settled after *t*_s_ = 1.1s. Lastly, the ADP-PI controller simulation, as displayed in Fig 7C settled at *t*_s_ = 1.0s to the reference trajectory compared to the mean settling time at ${\bar{t}}_{s}=2.3\text{s}$, while the open-loop simulation settled at ${\bar{t}}_{s}=1.1\text{s}$.

### Muscle contractions following sine input trajectories exhibit a lag response for all controllers and highly oscillatory behavior using the MRAC controller

The sine trajectories are shown in Fig 9A–9C. The experimental and simulation responses using the PI controller, shown in Fig 9A, exhibited some oscillatory behavior during relaxation at interval sets [3s, 5s] and [8s, 10s] as opposed to the open-loop simulation. The PI simulation was within bounds of the experimental trajectories while the open-loop case did not remain within the bounds during the initial contraction. The oscillations during muscle relaxation were attributed to the extension spring in the setup. This effect likely indicated that the spring force after muscle contraction was pulling the muscle faster than the time required for the muscle to return to its original length after stimulation. The MRAC sine trajectories from simulation and experiment were also characterized by oscillatory behavior during adaptation, as shown in Fig 9B. Both responses followed the set reference trajectory as opposed to the open-loop case, which was characterized by a higher amplitude and did not capture these fluctuations. Lastly, the sine trajectories using the ADP-PI controller displayed in Fig 9C, did not exhibit fluctuations as compared to the MRAC results. These trajectories followed the reference system in both experiment and simulation, but lagged after 3s of stimulation.

### Muscle contractions following ramp input trajectories are characterized by a response delay for all controllers

Oscillations prevailed for the ramp trajectories in experiment and simulation as shown in Fig 10A–10C as well as a delayed response of approximately 1s for all controllers. For the PI controller (Fig 10A), the mean ramp response and distribution attenuated after 6.5s as evidenced by the *σ* bounds converging closer to the desired trajectory. The MRAC controller in experiment and simulation showed highly oscillatory behavior throughout the 10s of stimulation as observed in Fig 10B. On the contrary, the ramp trajectories using the ADP-PI controller in experiment and simulation (refer to Fig 10C) attenuated its oscillatory behavior after 5.5s of stimulation, which could be attributed to the adaption component of the system.

### Muscle contractions following square input trajectories present a delay sustaining full contraction for all controllers

The square trajectories are illustrated in Fig 11A–11C. Stiction was observed for the square trajectories in simulation and experiment using the PI and ADP-PI controllers as shown in Fig 11A and 11C. The experimental and simulations using the MRAC controller sustained oscillatory behavior throughout the 9s of stimulation as observed in Fig 11B. This behavior was evident through the dispersion of the mean as shown by the *σ* bounds during contraction. Moreover, we observed that no experimental samples restored to its original position after the initial contraction. This behavior could be an indication of muscle fatigue and friction between the mass and rail of the setup. In addition, the closed-loop simulations presented similar behavior as the experiments as opposed to the open-loop simulation which did not capture the nonlinearities affecting the system.

### EDL muscle response to sine and ramp trajectories is significantly different across the closed-loop controllers

To evaluate the treatment of the controllers for muscle stimulation in experiment, the mean STE was used to test the following hypotheses: (1) the condition of the controller is significant and has an effect on muscle stimulation, and (2) the ADP-PI controller is the optimal controller to implement for muscle stimulation compared to PI and MRAC.

**Fig 12:**
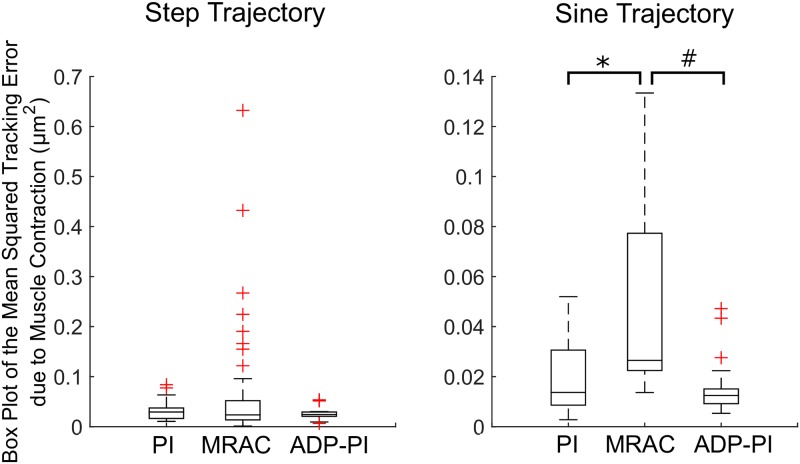
Box plots of the mean Squared Tracking Error (STE) due to EDL muscle contraction for the step and sine input trajectories. Statistical significance is denoted by *, # (*p* < 0.05).

To assess controller tracking performance, we analyzed the mean STE values between the three controllers for all trajectories. Box plots of the step and sine trajectories are shown in Fig 12. For the step trajectories, normality condition was satisfied from observations of the Q-Q plot of the residuals and we tested the means with Welch’s unequal variances t-test. There was moderate evidence suggesting the PI (*x*_*μ*_ ± *σ*, 0.031 ± 0.02), MRAC (0.066 ± 0.11), and ADP-PI (0.028 ± 0.01) controllers means were equal (*F*(2, 65.8) = 2.81, *p* = 0.06). A pair-wise comparison using Games-Howell post-hoc test revealed moderate evidence of differences between the PI and MRAC controllers (Mean Difference (MD) = 0.034, *p* = 0.09) and MRAC and ADP-PI controllers (MD = 0.037, *p* = 0.06). No significant difference was observed between PI and ADP-PI controllers (MD = 0.003, *p* = 0.77).

The sine trajectories satisfied the normality condition. Welch’s unequal variances t-test showed a strong evidence of a difference among the means of the PI (0.020 ± 0.02), MRAC (0.049 ± 0.04), and ADP-PI (0.015 ± 0.01) controllers ((*F*(2, 23.8) = 5.14, *p* = 0.01). A pair-wise comparison via Games-Howell post-hoc test reported significant differences between the PI and MRAC controllers (MD = 0.030, *p* = 0.04) and between the MRAC and ADP-PI controllers (MD = 0.034, *p* = 0.02). No significance was observed between PI and ADP-PI controllers (MD = 0.005, *p* = 0.62).

**Fig 13:**
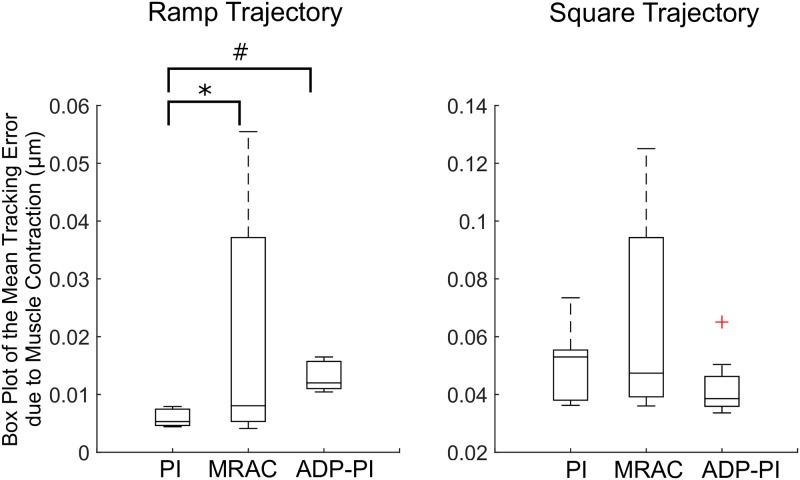
Box plots of the mean tracking error (STE) due to EDL muscle contraction for the ramp and square input trajectories. Statistical significance is denoted by *, # (*p* < 0.05).

For the ramp and square trajectories, box plots are displayed in Fig 13. For these trajectories, we performed a square root transformation to ensure normality, which was validated through Q-Q plots of the residuals. For the ramp trajectories, the Welch’s unequal variances t-test showed strong evidence of the difference in means (*F*(2, 15.7), *p* > 0.0001) for the PI (0.076 ± 0.01), MRAC (0.129 ± 0.07), and ADP-PI (0.114 ± 0.01) controllers. Through a pair-wise comparison using Games-Howell post-hoc test, significance was observed between the PI and MRAC controllers (MD = 0.053, *p* = 0.04) and the PI and ADP-PI controllers (MD = 0.038, *p* = 0.003). There was no significance between MRAC and ADP-PI controllers (MD = 0.014, *p* = 0.73). Lastly, the square trajectories satisfied the normality condition. The Welch’s unequal variances t-test presented moderate evidence of difference in means (*F*(2, 15.3) = 3.09, *p* = 0.07) for the PI (0.222 ± 0.03), MRAC (0.246 ± 0.06), and ADP-PI (0.204 ± 0.02) controllers. Through the Games-Howell post-hoc test no significant differences were observed between the PI and MRAC (MD = 0.023, *p* = 0.55), MRAC and ADP-PI (MD = 0.042, *p* = 0.17), and between PI and ADP-PI controllers (MD = 0.018, *p* = 0.19).

## Conclusions

Here, we have presented numerical simulations of muscle stimulation via adaptive and linear closed-loop controllers as well as experimental validation of these systems *ex vivo*. By testing the efficacy of the controllers using the EDL muscle in isolation, we can measure contractions solely due to the stimulation delivered by the control effort. The three feedback controllers are effective in tracking a variety of reference trajectories. In addition, the model simulated with the closed-loop controllers generally matched well with the experimental results, both in terms of quantitative metrics and qualitative behavior. Overall, experiments validated controller simulations since it captured nonlinearities such as delays and friction behaviors, which were not present in the open-loop responses.

All closed-loop controllers worked in simulation to track signals. To build the simulations, linear dynamics were assumed for muscle behavior while nonlinear controllers, with the exception of the PI controller and open-loop case, were applied. The successful outcomes of this comparative study highlights the advantage of feedback controllers in electrical stimulation without the requirements of higher order muscle models that carry great computational demands. Nevertheless, challenges still persist when implementing control systems for FES treatments, such as the nonlinearities of the muscle system, appropriate coordination of muscle group activation, non-physiological recruitment eliciting fatigue associated with muscle contractions, time delays of the biochemical processes between stimulation and the start of muscle contraction, spasticity, just to mention a few [12, 16, 43–46]. As pointed out by Narendra and Annaswammy [46], handling of biological systems, such as skeletal muscles, requires adaptation and understanding of the input/output relationship for the design of the controllers.

All closed-loop controllers are consistent with experiments. The closed-loop muscle system simulations qualitatively captured the behavior of the EDL mouse muscle as evidenced by the trajectory outputs in the Results section. Several assumptions were made when building, testing and coding the experimental system, which included Coulomb friction and no moment effects, among others. Moreover, spring reaction forces and friction contributed to the *stick-slip* behavior observed in all trajectories during muscle relaxation. These effects observed in experiments were captured by the closed-loop controllers in both simulation and experiments, as opposed to the open-loop cases.

The ADP-PI controller system has the best tracking performance compared to that of the PI and MRAC controller. The rate of muscle fiber contraction and adaptation worked in synergy to output adequate tracking of the reference system with minimal system error. Although ten parameters characterized the system, the linear and nonlinear components could be tuned separately for optimal output. Thus, the ADP-PI controller stimulated the muscle efficiently by providing real-time changes to the gains and by reducing the influence of perturbations in the system (e.g. small losses in muscle force over the course of the experiment). Overall, adaptation enabled fastest tracking error convergence and remained stable while the parameters varied in time. The ADP-PI controller best tracked the set trajectories by first using the PI component to approach the area of convergence, thus minimizing the system error while the adaptive component fine-tuned the controller. In this way, trajectory responses were characterized by minimal oscillatory behavior, as compared to the MRAC controller which lacks this synergy.

Closed-loop systems are capable of using the feedback information to minimize the system error and tracking of muscle contraction. However, comparing the closed-loop controllers in experiments through statistical analyses revealed a significant effect of controller type on muscle stimulation for the sine and ramp trajectory inputs. As shown in Figs 12–13, mean squared tracking error was significantly different between the PI and MRAC as well as the MRAC and ADP-PI for the sine trajectories and the PI and MRAC as well as the PI and ADP-PI for the ramp trajectories. Overall, the ADP-PI had the smallest variance overall. The PI controller had a smaller variance compared to MRAC. On the other hand, MRAC showed faster adaptation compared to the rate of muscle fiber activation as characterized by overshoot and oscillations captured for both simulation and experiment. By incorporating the linear and adaptive components via the ADP-PI controller, we were able to apply a robust feedback system capable of handling system uncertainties and unmodeled dynamics. We conclude that ADP-PI controller offers the best performance due to its incorporation of the linear and adaptive components of the PI and MRAC systems.

From a theoretical perspective, closed-loop controllers mimic the feedback inherent in the nervous system, since our nerves carry out both commands to muscles and feedback to the central nervous system; thus closed-loop control may be better integrated with, biological systems. The open-loop approach is not a natural process and simulations for this case failed to capture the key characteristics observed in our FES experiments. Although the same muscle-mass-spring system was applied for all simulations, the open-loop case was unable to assess the muscle response through tracking and capture key behaviors, such as friction and response delays. Conversely, tuning capabilities through closed-loop systems have the ability to capture these behaviors and compensate for muscle fatigue by changing the control input and outputting the appropriate PWM signal.

Nevertheless, these controllers warrant further investigation, for example, to study the mismatch between the experimentally-observed control effort and that predicted by the model. Changes in the control effort are displayed in the Supporting Information section in S2–S7 Figs. We observed that the experimental control effort was generally smaller for experiments than predicted by simulations. During experiments, the muscle was suspended in a bath submerged in ionized solution which was not represented in simulation. The differences between these control input signals and muscle response will be explored in future studies.

In conclusion, the application of feedback controllers, very rarely used in FES applications, has the potential to become an effective tool for treatments regarding skeletal muscle stimulation. Adaptive controllers, especially the ADP-PI controller, provide a beneficial strategy for muscle treatment by providing appropriate stimulation to the affected muscle and considering the muscle response to set stimulation. This *proof of principle* can be extrapolated to a clinical setting as a rehabilitation tool since it can provide patients with an efficient method of muscle activation. From a translational research perspective, next steps involve two major components: (1) medical device design to incorporate these controllers in a user-friendly rehabilitation tool comprising of electrodes, stimulator, and embedded systems tracking joint movement, and (2) standard input trajectories for tracking joint movement to ensure best practices for patient rehabilitation. Both aspects introduce new challenges to guarantee reliable medical device design, ensure safety procedures, and integrate human factors for future clinical trials.

## Supporting information

S1 Table Nomenclature. (PDF)

S1 Appendix Skeletal muscle model. (PDF)

S2 Table Thelen muscle parameters for the skeletal muscle model. These parameters were used for simulations of the EDL mouse muscle model.
(PDF)

S2 Appendix Tuning of controller procedure. (PDF)

S1 Fig Various step trajectory responses due to EDL muscle contraction using the PI controller. Different values for *k*_P_ and *k*_I_ are determined to identify the appropriate tuning parameters.
(PDF)

S3 Table Timeline of muscle experiment. A typical cascade of events when carrying out an experiment.
(PDF)

S2 Fig Samples of the step and sine pulse duration inputs and trajectory outputs using the PI controller. Experimental sample applying the PI controller for muscle contraction compared to the muscle system simulation for the PI and open-loop case. Trajectories include (A) the step and (B) sine functions.
(TIF)

S3 Fig Samples of the ramp and square pulse duration inputs and trajectory outputs using the PI controller. Experimental sample applying the PI controller for muscle contraction compared to the muscle system simulation for the PI and open-loop case. Trajectories include (C) ramp and (D) square functions.
(TIF)

S4 Fig Samples of the step and sine pulse duration inputs and trajectory outputs using the MRAC controller. Experimental sample applying the MRAC controller for muscle contraction compared to the muscle system simulation for the MRAC and open-loop case. Trajectories include (A) the step and (B) sine functions.
(TIF)

S5 Fig Samples of the ramp and square pulse duration inputs and trajectory outputs using the MRAC controller. Experimental sample applying the MRAC controller for muscle contraction compared to the muscle system simulation for the MRAC and open-loop case. Trajectories include (C) ramp and (D) square functions.
(PDF)

S6 Fig Samples of the step and sine pulse duration inputs and trajectory outputs using the ADP-PI controller. Experimental sample applying the ADP-PI controller for muscle contraction compared to the muscle system simulation for the ADP-PI and open-loop case. Trajectories include (A) the step and (B) sine functions.
(TIF)

S7 Fig Samples of the ramp and square pulse duration inputs and trajectory outputs using the ADP-PI controller. Experimental sample applying the ADP-PI controller for muscle contraction compared to the muscle system simulation for the ADP-PI and open-loop case. Trajectories include (C) ramp and (D) square functions.
(TIF)
