# Supplementary material for: Classical and adaptive control of ex vivo skeletal muscle contractions using Functional Electrical Stimulation (FES)
Source: PLoS One. 2017 Mar 8;12(3):e0172761. doi: 10.1371/journal.pone.0172761 (PMC5342220; doi:10.1371/journal.pone.0172761)
Supplement: S1 Table — (PDF) [file pone.0172761.s001.pdf]

S1 Table. Nomenclature describing definitions of variables.

a	Unknown state constant
a_{r}	Reference model state constant
A_{r}	Known state matrix
b	Unknown input constant of known sign
b_{r}	Reference model input constant
c	Damping coefficient
$e_{\mathrm{ad}}(t)$	Error of $x(t)$ relative to $x_{\mathrm{r}}(t)$ for MRAC
$e_{\mathrm{PI}}(t)$	Error of $x(t)$ relative to $x_{\mathrm{d}}(t)$ for PI Control
$e_{\mathrm{a}}(t)$	Error dynamics of the Adaptive Augmented PI Controller
F_{f}	Coulomb frictional force
F^{MT}	Muscle-tendon force
γ_x, γ_{r}	MRAC tuning parameter constants
Γ_K,Γ_W	Adaptive Augmented PI Controller adaptation gains
k_{I}	Integral Gain for PI Control
k_{P}	Proportional Gain for PI Control
K^*	Matrix containing integral and proportional gains for PI Control
$\tilde{K}(t)$	Approximation error between the estimate and ideal values of K
$\dot{\hat{K}}(t)$	Estimate of K
k_{s}	Spring constant
l_{M}	Muscle length
l_0^{M}	Maximum optimal fiber length at resting tension
l_{MT}	Muscle-tendon length
$l_{\mathrm{s}}^{\mathrm{T}}$	Tendon slack length
λ^*	Constant of known sign and unknown magnitude
m	Mass of the setup
MRAC	Model Reference Adaptive Control
P	Positive definite matrix that satisfies the Lyapunov equation
PI	Proportional Integral
$\phi(x(t))$	Known vector of nonlinear functions
$\mathrm{r}(t)$	Reference Input
T_{i}	Integral Time

θ_x^*, θ_r^*	Ideal controller gains for MRAC
$\theta_x(t)$, $\theta_\mathrm{r}(t)$	Adaptive controller gain for MRAC
$\tilde{\theta}_x(t)$, $\tilde{\theta}_\mathrm{r}(t)$	Difference between adaptive and ideal gain
$u(t)$	Control effort
$u_\mathrm{ad}(t)$	MRAC control input effort
$u_\mathrm{id}(t)$	Ideal MRAC control input effort
$u_\mathrm{PI}(t)$	PI control input effort
$V(t)$	Lyapunov function
W^*	Vector of nonlinear function weights
$\tilde{W}(t)$	Approximation error between the estimate and ideal values of W
$\dot{\hat{W}}(t)$	Estimate of W
$x(t)$	System state
$x_\mathrm{a}(t)$	Augmented system state
$x_\mathrm{d}(t)$	Desired Trajectory
$x_\mathrm{I}(t)$	Integral state of PI Control
$x_\mathrm{lin}(t)$	Reference model state for Adaptive Augmented PI controller
$x_\mathrm{r}(t)$	Reference model state for MRAC
