# Supplementary material for: Classical and adaptive control of ex vivo skeletal muscle contractions using Functional Electrical Stimulation (FES)
Source: PLoS One. 2017 Mar 8;12(3):e0172761. doi: 10.1371/journal.pone.0172761 (PMC5342220; doi:10.1371/journal.pone.0172761)
Supplement: S1 Appendix — (PDF) [file pone.0172761.s002.pdf]

S1 Appendix. Skeletal Muscle Model.

The muscle model requires an input activation which results into muscle force. The inputs are the excitation ($e(t)$) and activation dynamics ($a(t)$), the muscle-tendon length l_{MT}, optimal muscle fiber length l_0^{M}, optimal muscle force F_0^{M}, and two fatigue parameters. The model assumes a zero pennation angle ($\alpha = 0$). The muscle length, l_{M}, and tendon slack length, $l_{\mathrm{s}}^{\mathrm{T}}$, comprises l_{MT}. These parameters and others used throughout this section are specified on Table S1. The other parameters in the model are the same across all skeletal muscles as detailed by [S1]. A bar over the variables representing lengths indicates normalization with respect to l_0^{M}. Likewise, a bar over variables representing forces indicate normalization with respect to F_0^{M}.

Once the trajectory is specified, the controllers generate a pulse duration signal as described in the Controller Setup section with an upper limit of 200μs. The pulse duration sets on an excitation, $e(t)$, with values ranging from 0 to 1. Excitation is related to muscle activation, $a(t)$ with values also ranging from 0 to 1. The activation dynamics is then determined as

$$\dot{a}(a(t), e(t)) = \begin{cases} \dfrac{e(t) - a(t)}{\tau_{act}}, & e(t) \geq a(t), \\ \\ \dfrac{e(t) - a(t)}{\tau_{deact}}, & e(t) < a(t) \end{cases}$$

where $\tau_{act} = 0.01$s and $\tau_{deact} = 0.05$s. The activation and deactivation times were based on EDL mouse muscle studies conducted by [S2]. There is zero activation at $t = 0$.

The tendon length (l_{T}) and corresponding tendon force (F^{T}) are calculated through the relations

$$l_{\mathrm{T}} = l_0^{\mathrm{M}} + l_{\mathrm{s}}^{\mathrm{T}} - l_{\mathrm{M}}, \text{ and} \tag{1}$$

$$\bar{F}^{T} = \frac{(1 + \varepsilon^{\mathrm{T}})}{1000} + \begin{cases} k_{\mathrm{lin}}(\varepsilon^{\mathrm{T}} - \varepsilon^{\mathrm{T}}_{\mathrm{toe}}) + \bar{F}^{\mathrm{T}}_{toe}, & \varepsilon^{\mathrm{T}} > \varepsilon^{\mathrm{T}}_{\mathrm{toe}} \\ F_{\mathrm{toe}} \frac{e^{k_{\mathrm{toe}}\varepsilon^{\mathrm{T}}/\varepsilon^{\mathrm{T}}_{\mathrm{toe}}-1}}{e^{k}_{\mathrm{toe}}-1}, & 0 < \varepsilon^{\mathrm{T}} \leq \varepsilon^{\mathrm{T}}_{\mathrm{toe}} \\ 0. \end{cases} \tag{2}$$

The contraction force of the muscle is $F^{\mathrm{MT}}(t) = F^{\mathrm{T}}(t)$, since the contraction force of the muscle fibers is transferred to the tendon force. Moreover, passive force (F^{PE}) occurs when the muscle is being lengthened. The EDL muscle is set to l_0^{M} at $t = 0$, therefore, the muscle is in a passive state. The passive muscle strain, $\varepsilon_0^{\mathrm{M}}$, is set at 0.6 while the exponential factor shape for the force-length relationship, k_{PE} is set at 4. F^{PE} in the muscle fibers is calculated as: $F^{\mathrm{PE}}{=}F_0^{\mathrm{M}}\bar{F}^{\mathrm{PE}}$. The normalized passive force, $\bar{F}^{\mathrm{PE}}$, is given by the relation

$$\bar{F}^{\mathrm{PE}}(\bar{l}_0^{\mathrm{M}}(t)) = \begin{cases} 1 + \dfrac{k_{\mathrm{PE}}}{\varepsilon_0^{\mathrm{M}}}(\bar{l}_0^{\mathrm{M}} - (1 + \varepsilon_0^{\mathrm{M}})), & \bar{l}_0^{\mathrm{M}} > 1 + \varepsilon_0^{\mathrm{M}} \\ \\ \dfrac{e^{k_{\mathrm{PE}}(\bar{l}_{\mathrm{M}}-1)/\varepsilon_0^{\mathrm{M}}}}{e^{k_{\mathrm{PE}}}}, & \bar{l}_{\mathrm{M}} \leq 1 + \varepsilon_0^{\mathrm{M}}. \end{cases} \tag{3}$$

The contractile element force, F^{CE}, is calculated by the difference between the tendon force, F_{T} and the passive force F^{PE}. That is, $F^{\mathrm{CE}}(t) = F^{\mathrm{T}}(t) - F^{\mathrm{PE}}(t)$ since we

are assuming the pennation angle is zero. Moreover, the active force of the muscle fibers, F^{a}, is a function of the activation a, normalized muscle fiber, $\bar{l}_{\mathrm{M}}$, and a Gaussian function representing the normalized F-L relationship, f_l,

$$F^{\mathrm{a}} = a(t)F_0^{\mathrm{M}} f_l, \quad \text{where} \quad f_l = \frac{e^{-(\bar{l}_{\mathrm{M}}-1)^2}}{\gamma}. \tag{4}$$

The contraction dynamics are calculated as explained through the work detailed in [S1] by first determining the force-velocity scale factor, $(\bar{F}_{\mathrm{V}}^{\mathrm{M}})^{-1}$, and the contraction velocity of the muscle fibers, $\dot{l}_{\mathrm{M}}$. $(\bar{F}_{\mathrm{V}}^{\mathrm{M}})^{-1}$ is a function of F^{CE} and F^{a}. Additional terms are included such as a passive damping factor, $\xi = 0.05$, and $\varepsilon = 10^{-6}$ to avoid numerical errors in the computation.

The $(\bar{F}_{\mathrm{V}}^{\mathrm{M}})^{-1}$ equation is given by

$$(\bar{F}_{\mathrm{V}}^{\mathrm{M}})^{-1}(F^{\mathrm{CE}}, F^{\mathrm{a}}) = \begin{cases} \dfrac{F^{\mathrm{CE}}}{\varepsilon}\left(\dfrac{\varepsilon - F^{\mathrm{a}}}{F^{\mathrm{a}} + \varepsilon/A_{\mathrm{f}} + \xi} + \dfrac{F^{\mathrm{a}}}{F^{\mathrm{a}} + \xi}\right) - \dfrac{F^{\mathrm{a}}}{F^{\mathrm{a}} + \xi}, & F^{\mathrm{CE}} < 0 \\ \dfrac{F^{\mathrm{CE}} - F^{\mathrm{a}}}{F^{\mathrm{a}} + F^{\mathrm{CE}}/A_{\mathrm{f}} + \xi}, & 0 \le F^{\mathrm{CE}} < F^{\mathrm{a}} \\ \dfrac{F^{\mathrm{CE}} - F^{\mathrm{a}}}{\dfrac{(2 + 2/A_{\mathrm{f}})(F^{\mathrm{a}}\bar{F}_{\mathrm{len}}^{\mathrm{M}} - F^{\mathrm{CE}})}{\bar{F}_{\mathrm{len}}^{\mathrm{M}} - 1} + \xi}, & F^{\mathrm{a}} \le F^{\mathrm{CE}} < 0.95F^{\mathrm{a}}\bar{F}_{\mathrm{len}}^{\mathrm{M}} \\ f_{v_0} + \dfrac{F^{\mathrm{CE}} - 0.95F^{\mathrm{a}}\bar{F}_{\mathrm{len}}^{\mathrm{M}}}{\varepsilon F^{\mathrm{a}}\bar{F}_{\mathrm{len}}^{\mathrm{M}}}(f_{v_1} - f_{v_0}), & 0.95F^{\mathrm{a}}\bar{F}_{\mathrm{len}}^{\mathrm{M}} \le F^{\mathrm{CE}} \end{cases}$$

where f_{v_0} and f_{v_1} are defined as,

$$f_{v_0} = \frac{0.95F^{\mathrm{a}}\bar{F}_{\mathrm{len}}^{\mathrm{M}} - F^{\mathrm{a}}}{\dfrac{(2 + 2/A_{\mathrm{f}})0.05F^{\mathrm{a}}\bar{F}_{\mathrm{len}}^{\mathrm{M}}}{\bar{F}_{\mathrm{len}}^{\mathrm{M}} - 1} + \xi}, \tag{5}$$

$$f_{v_1} = \frac{(0.95 + \varepsilon)F^{\mathrm{a}}\bar{F}_{\mathrm{len}}^{\mathrm{M}} - F^{\mathrm{a}}}{\dfrac{(2 + 2/A_{\mathrm{f}})(0.05 - \varepsilon)F^{\mathrm{a}}\bar{F}_{\mathrm{len}}^{\mathrm{M}}}{\bar{F}_{\mathrm{len}}^{\mathrm{M}} - 1} + \xi}. \tag{6}$$

The muscle fiber velocity is determined by the contraction velocity as follows,

$$\dot{l} = V_{\max}(\bar{F}_{\mathrm{V}}^{\mathrm{M}})^{-1}\frac{F^{\mathrm{CE}}}{F^{\mathrm{a}}}, \text{where} \tag{7}$$

$$V_{\max} = (5 + 5a(t))l_0^{\mathrm{M}}. \tag{8}$$

A fatigue component was added to the system to better characterize the response of EDL muscles' fast-twitch type IIA and IIB fibers [S3–5]. The fatigue model adapted is proposed by Liu and Brown et al. [S6]. Muscle fatigue occurs over time due to the lack of activation of muscle fibers to contract. The fatigue model is based on compartmentalized motor units which includes the muscle fibers and the motor neurons that it innervates. The model divides these motor units into three groups: (1) activated compartment motor units, $M_{\mathrm{A}}(t)$, which exert the maximum force; (2) fatigued compartment motor units, $M_{\mathrm{F}}(t)$; and (3) resting compartment motor units, $M_{\mathrm{R}}(t)$.

The available contractile force is limited by the motor units available for force production, that is, activated and resting compartments. The residual capacity of the muscle, $\beta(t)$, introduced by Silva et al. [S7] is defined as

$$\beta(t) = M_{\mathrm{A}}(t) + M_{\mathrm{R}}(t) = 1 - M_{\mathrm{F}}(t). \tag{9}$$

The fatigued muscle force will then be given by

$$F_{\mathrm{f}}^{\mathrm{T}}(t) = F^{\mathrm{T}}(t)\beta(t), \tag{10}$$

which is the force of the system in our setup. Moreover, the three main equations that describe the fatigue model are as follows [S6]

$$M_{\mathrm{R}} = M_{\mathrm{O}} - M_{\mathrm{A}} - M_{\mathrm{F}}, \tag{11}$$

$$\dot{M}_{\mathrm{A}} = \beta(t)M_{\mathrm{R}} - \phi M_{\mathrm{A}} + \rho M_{\mathrm{F}}, \qquad M_{\mathrm{A}}(0) = 0, \tag{12}$$

$$\dot{M}_{\mathrm{F}} = \beta M_{\mathrm{A}} - \rho M_{\mathrm{F}}, \qquad M_{\mathrm{F}}(0) = 0. \tag{13}$$

The parameters ϕ, ρ are the fatigue factor and the recovery factor, respectively. $\beta(t)$ represents the activation generated by the open-loop and closed-loop controllers, $u(t)$. The total number of available motor units, M_{O} is 25 for the EDL mouse muscle.

Supporting Information References

S1. John, C. T., Anderson, F. C., Higginson, J. S., & Delp, S. L. (2013) Stabilisation of Walking by Intrinsic Muscle Properties Revealed in a Three-Dimensional Muscle-Driven Simulation. Computer Methods in Biomechanics and Biomedical Engineering. 16: 451–462.

S2. Kosterina, N. (2012). Modeling of Muscular Force Induced by Non-Isometric Contraction. KTH Royal Institute of Technology.

S3. Brooks, S. V., & Faulkner, J. A. (1988) Contractile Properties of Skeletal Muscles from Young, Adult and Aged Mice. The Journal of Physiology. 404: 71–82.

S4. Klueber, K. M., Feczko, J. D., Schmidt, G., & Watkins, J. B. (1989) Skeletal Muscle in the Diabetic Mouse: Histochemical and Morphometric Analysis. The anatomical Record, 225: 41–45.

S5. Haida, N., Fowler Jr, W. M., Abresch, R. T., Larson, D. B., Sharman, R. B., Taylor, R. G., & Entrikin, R. K. (1989) Effect of Hind-Limb Suspension on Young and Adult Skeletal Muscle: I. Normal mice. Experimental Neurology. 103: 68–76.

S6. Liu, J. Z., Brown, R. W., & Yue, G. H. (2002). A Dynamical Model of Muscle Activation, Fatigue, and Recovery. Biophysical Journal, 82: 2344–2359.

S7. Silva, M. T., Pereira, A. F., & Martins, J. M. (2011) An Efficient Muscle Fatigue Model for Forward and Inverse Dynamic Analysis of Human Movements. Procedia IUTAM. 2: 262–274.
