# Supplementary material for: Classical and adaptive control of ex vivo skeletal muscle contractions using Functional Electrical Stimulation (FES)
Source: PLoS One. 2017 Mar 8;12(3):e0172761. doi: 10.1371/journal.pone.0172761 (PMC5342220; doi:10.1371/journal.pone.0172761)
Supplement: S2 Table — These parameters were used for simulations of the EDL mouse muscle model. (PDF) [file pone.0172761.s003.pdf]

S2 Table. Thelen Muscle Parameters for the Skeletal Muscle Model. These parameters were used for simulations of the EDL mouse muscle model.

Muscle Length	l_{M}	0.0117 m
Optimal isometric force	F_0^{M}	0.507 N
Maximum optimal fiber length	l_0^{M}	$0.44 l_M$ m
Tendon slack length	$l_{\mathrm{s}}^{\mathrm{T}}$	0.0129 m
Passive muscle strain	$\varepsilon_0^{\mathrm{M}}$	0.006
Passive F-L shape factor	k_{PE}	4.0
Exponential shape factor	k_{toe}	3
Tendon strain due to F_0^{M}	$\varepsilon_0^{\mathrm{T}}$	0.033
Linear shape factor	k_{lin}	$1.712/\varepsilon_0^{\mathrm{T}}$
Tendon strain (linear)	$\varepsilon_{\mathrm{toe}}^{\mathrm{T}}$	$0.609\varepsilon_0^{\mathrm{T}}$
Normalized tendon force (linear)	$\bar{F}_{\mathrm{toe}}^{\mathrm{T}}$	0.333333
Shape factor for the Gaussian active F-L	γ	0.5
Shape factor for the F-V	A_{f}	0.3
Normalized muscle force during lengthening	$\bar{F}_{\mathrm{len}}^{\mathrm{M}}$	1.8
