# Supplementary material for: Classical and adaptive control of ex vivo skeletal muscle contractions using Functional Electrical Stimulation (FES)
Source: PLoS One. 2017 Mar 8;12(3):e0172761. doi: 10.1371/journal.pone.0172761 (PMC5342220; doi:10.1371/journal.pone.0172761)
Supplement: S2 Appendix — (PDF) [file pone.0172761.s004.pdf]

S2 Appendix. Tuning of Controller Procedure.

For every muscle experiment, all trials started with a twitch response followed by a series of step input responses. All controllers require tuning of parameters; therefore through the step input we were able to determine the tuning parameters. For instance, the PI controller requires tuning of two gains: k_{P} and k_{I}. A method similar to Ziegler-Nichols was implemented to identify these gains. S1 Fig. shows several trials for the step input to determine the appropriate tuning parameters to follow the desired trajectory, x_{d}. First, an arbitrary k_{P} gain was specified with zero k_{I} value. The k_{P} gain was increased until oscillations were observed from the muscle contractions. Next, k_{P} amount was lowered and k_{I} parameter was introduced. The process continues until the muscle contractions are able to follow x_{d} and tracking error is minimized. Nevertheless, tuning was performed several times for the same muscle due to the decay of muscle function throughout different trials. Similarly, tuning of parameter methodology was conducted of the nonlinear controllers, MRAC and ADP-PI.
