# Supplementary figures and images for: Classical and adaptive control of ex vivo skeletal muscle contractions using Functional Electrical Stimulation (FES)
Source: PLoS One. 2017 Mar 8;12(3):e0172761. doi: 10.1371/journal.pone.0172761 (PMC5342220; doi:10.1371/journal.pone.0172761)

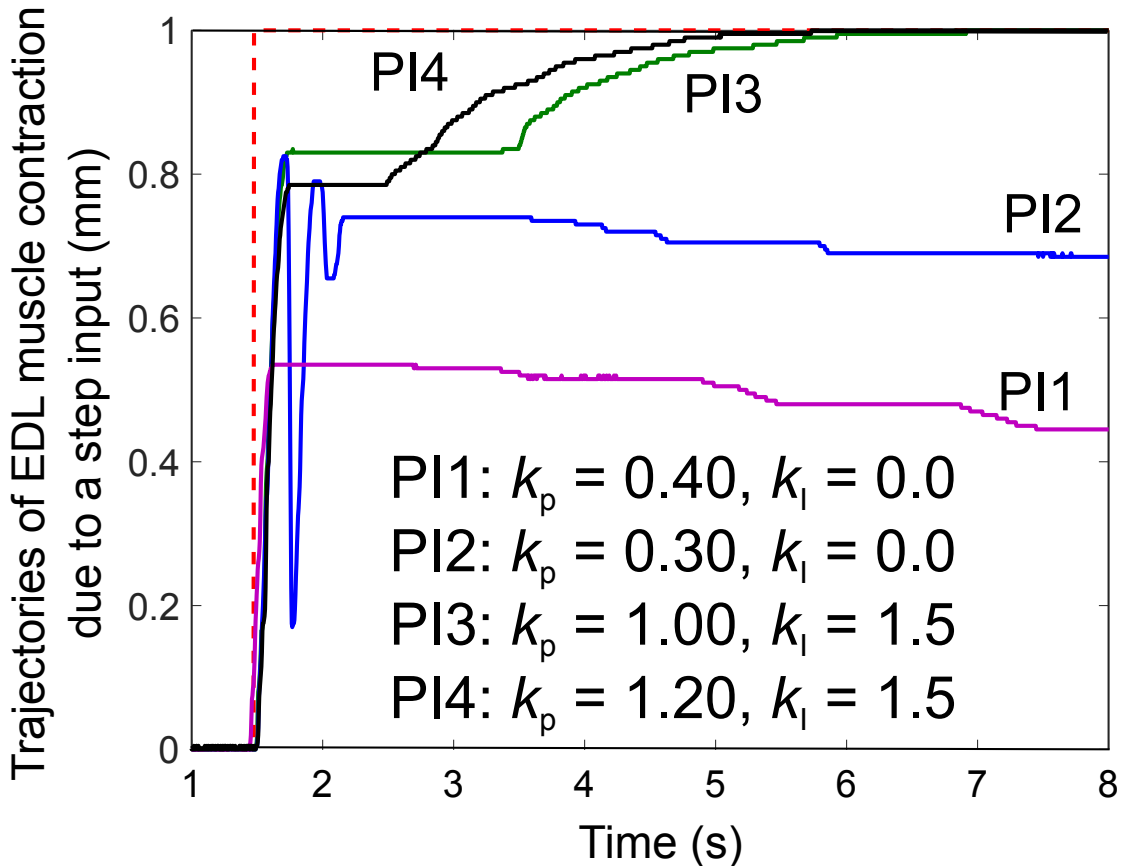

Supplement: S1 Fig — Different values for kP and kI are determined to identify the appropriate tuning parameters. (PDF) [file pone.0172761.s005.pdf]

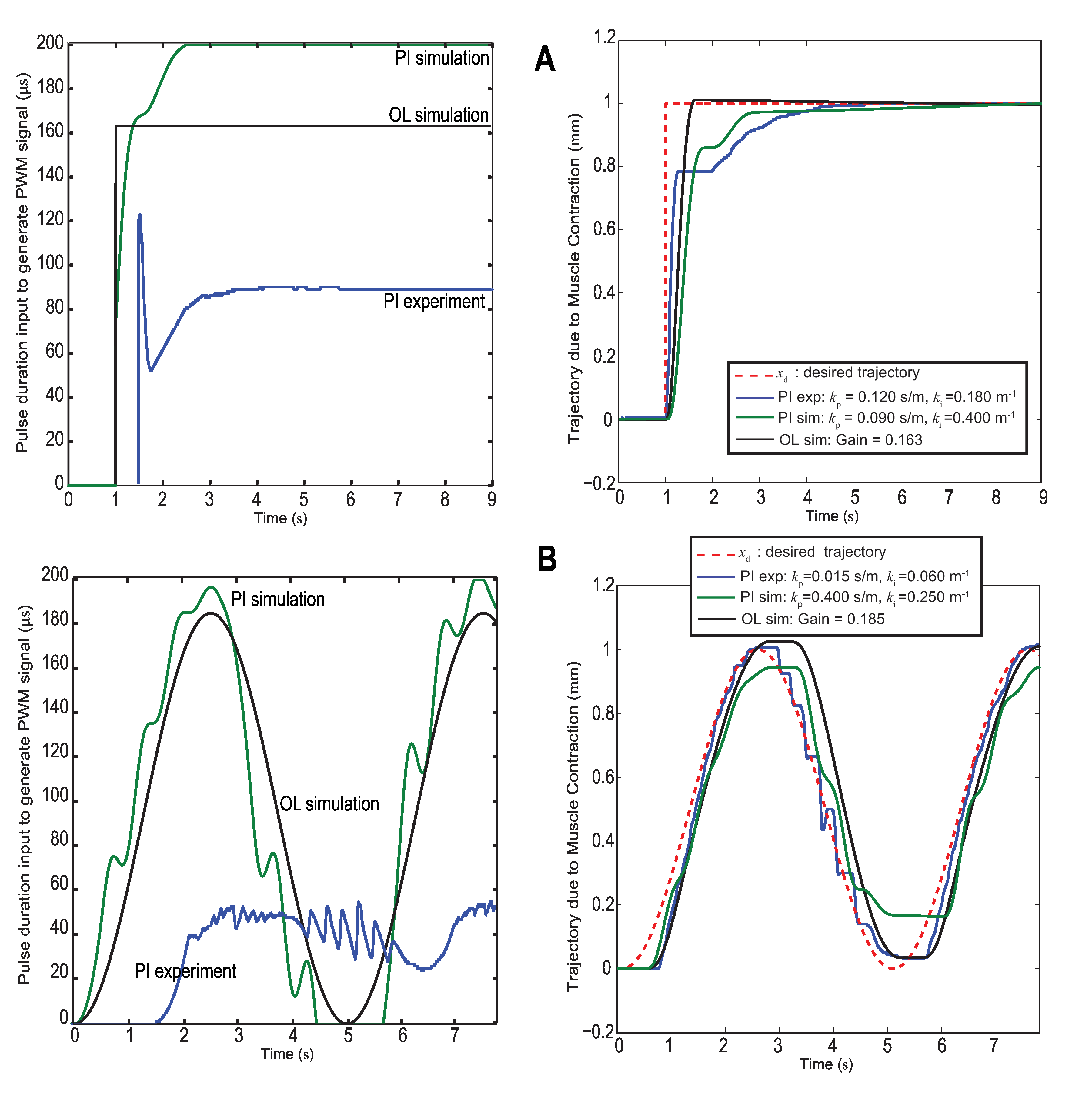

Supplement: S2 Fig — Experimental sample applying the PI controller for muscle contraction compared to the muscle system simulation for the PI and open-loop case. Trajectories include (A) the step and (B) sine functions. (TIF) [file pone.0172761.s007.tif]

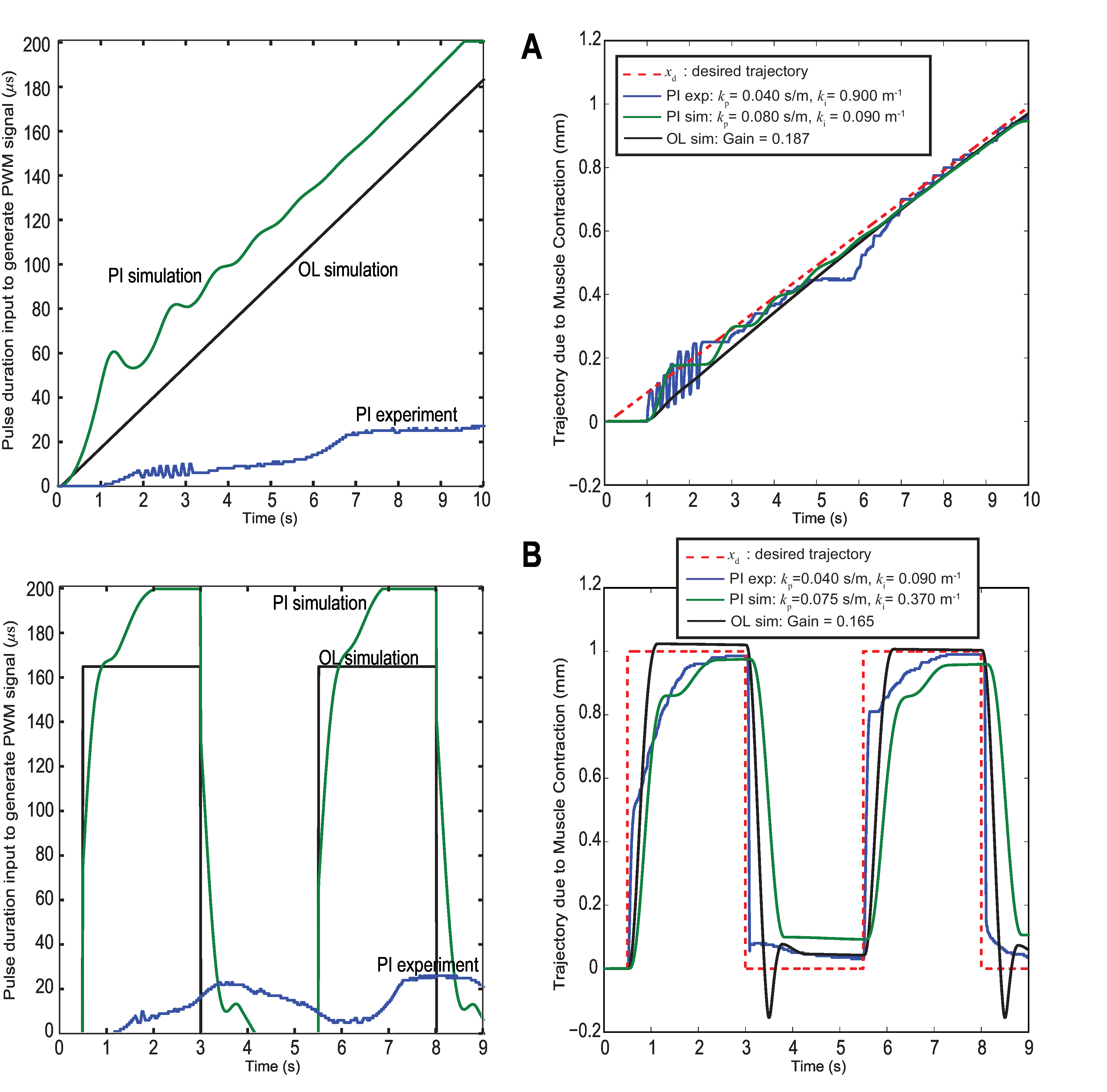

Supplement: S3 Fig — Experimental sample applying the PI controller for muscle contraction compared to the muscle system simulation for the PI and open-loop case. Trajectories include (C) ramp and (D) square functions. (TIF) [file pone.0172761.s008.tif]

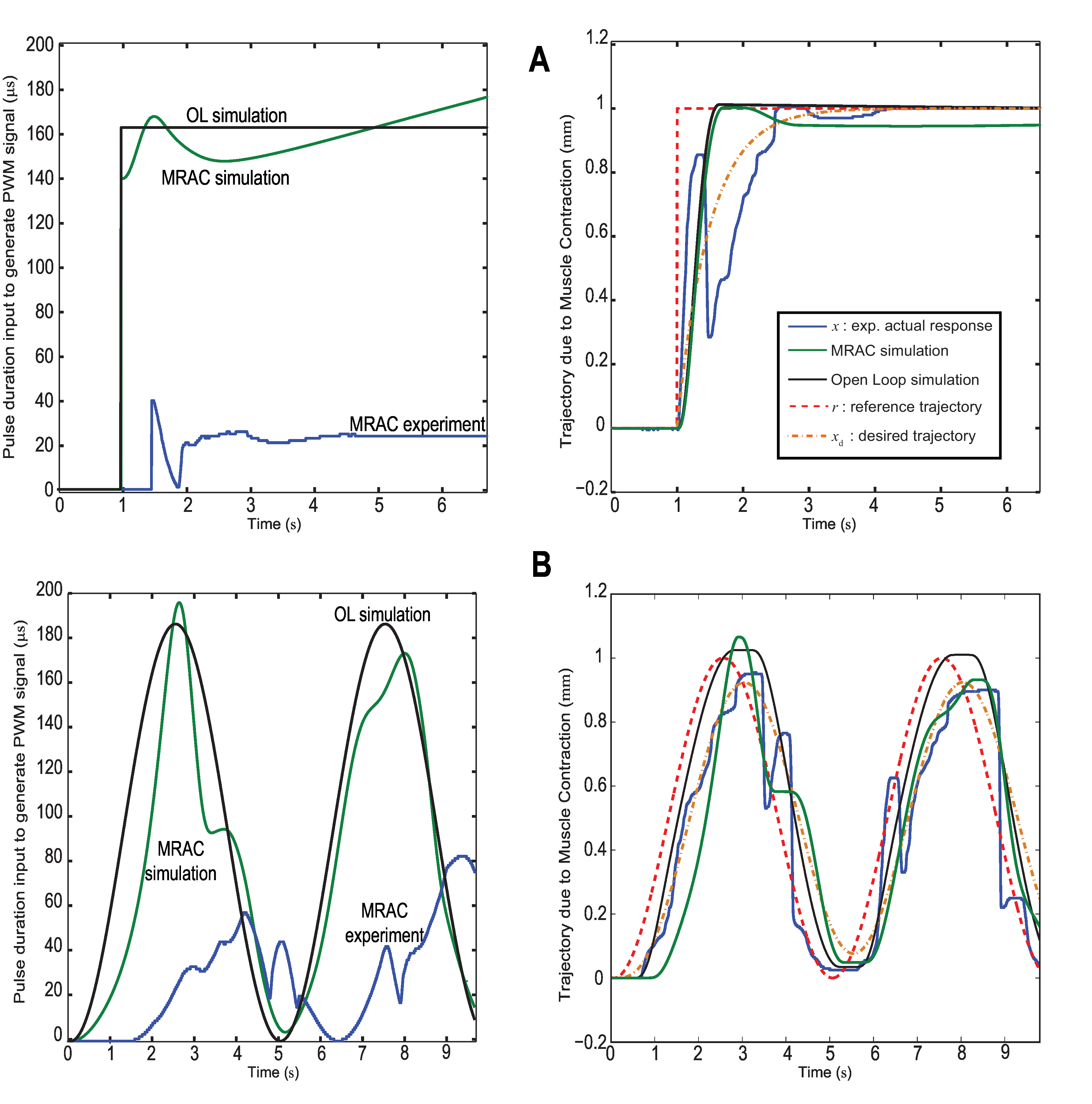

Supplement: S4 Fig — Experimental sample applying the MRAC controller for muscle contraction compared to the muscle system simulation for the MRAC and open-loop case. Trajectories include (A) the step and (B) sine functions. (TIF) [file pone.0172761.s009.tif]

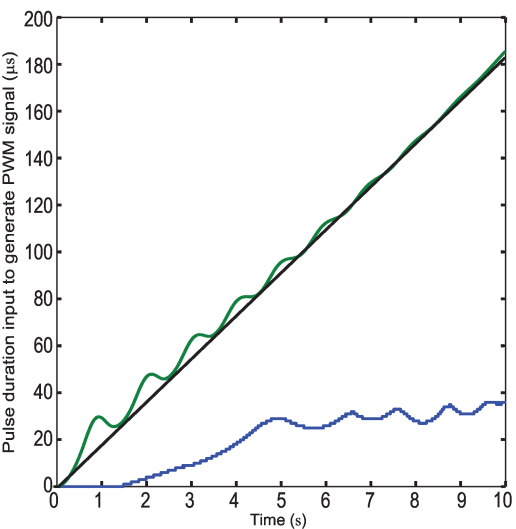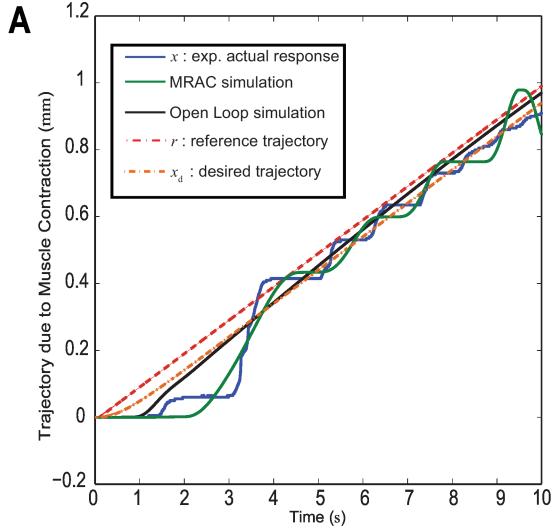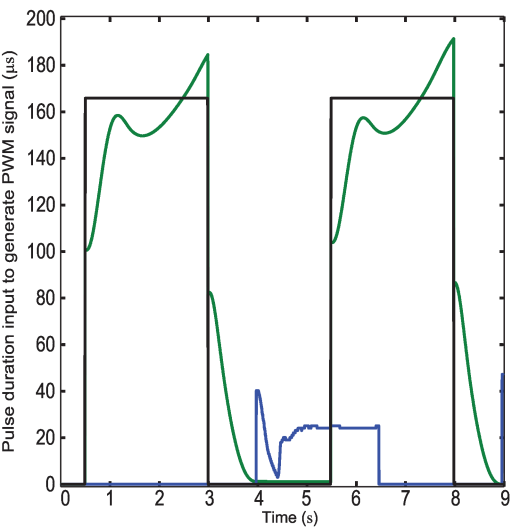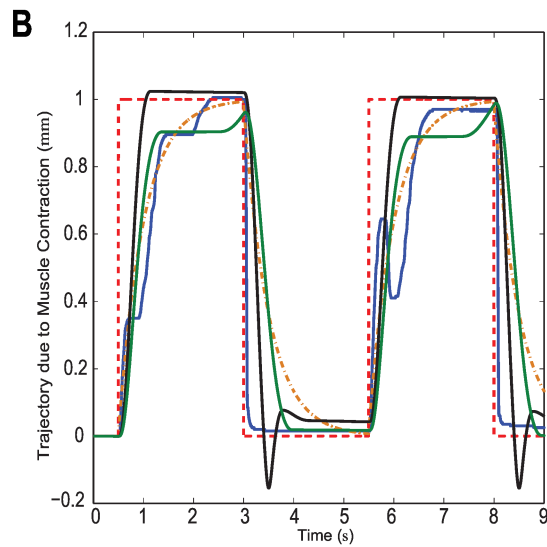

Supplement: S5 Fig — Experimental sample applying the MRAC controller for muscle contraction compared to the muscle system simulation for the MRAC and open-loop case. Trajectories include (C) ramp and (D) square functions. (PDF) [file pone.0172761.s010.pdf]

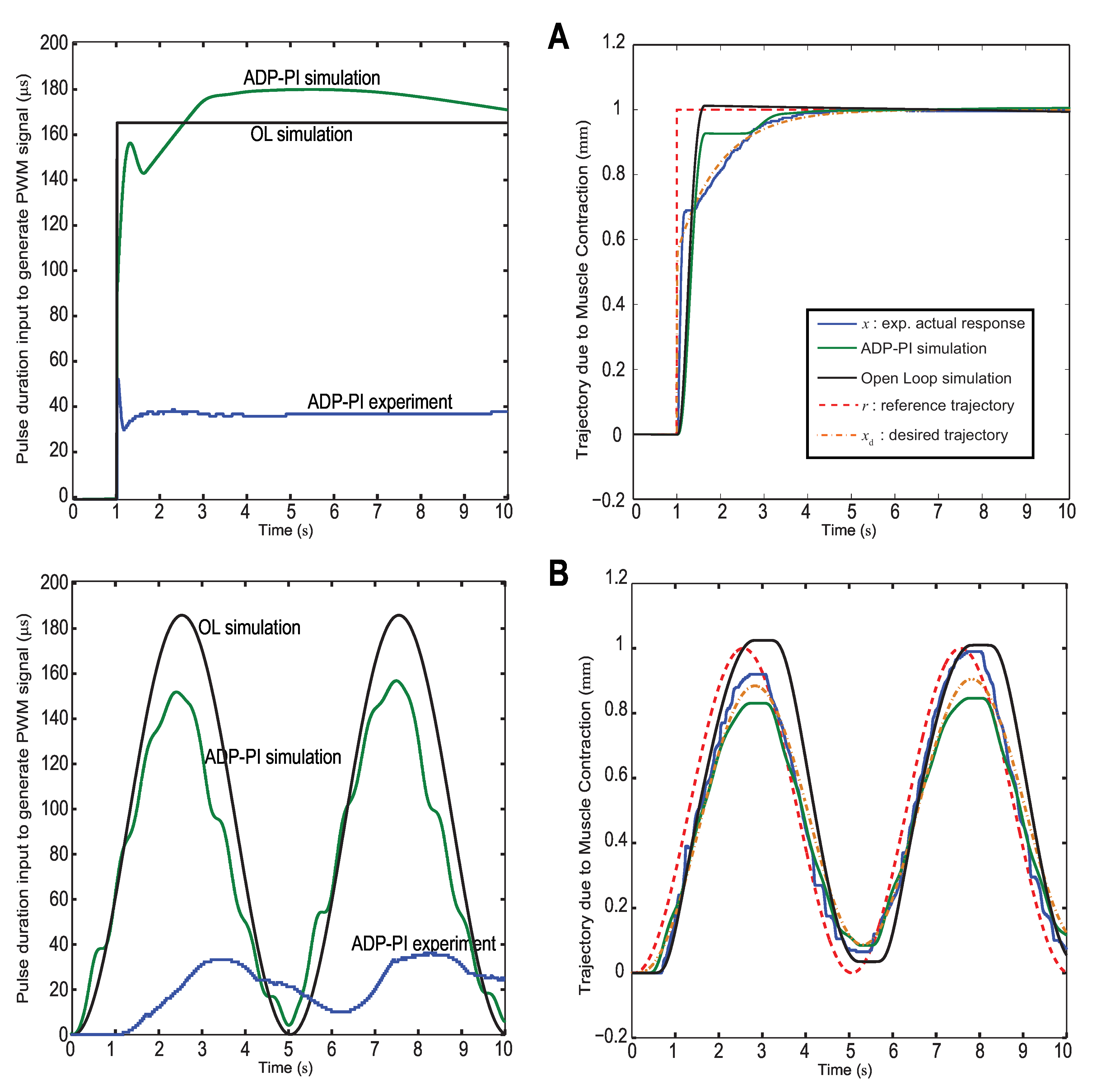

Supplement: S6 Fig — Experimental sample applying the ADP-PI controller for muscle contraction compared to the muscle system simulation for the ADP-PI and open-loop case. Trajectories include (A) the step and (B) sine functions. (TIF) [file pone.0172761.s011.tif]

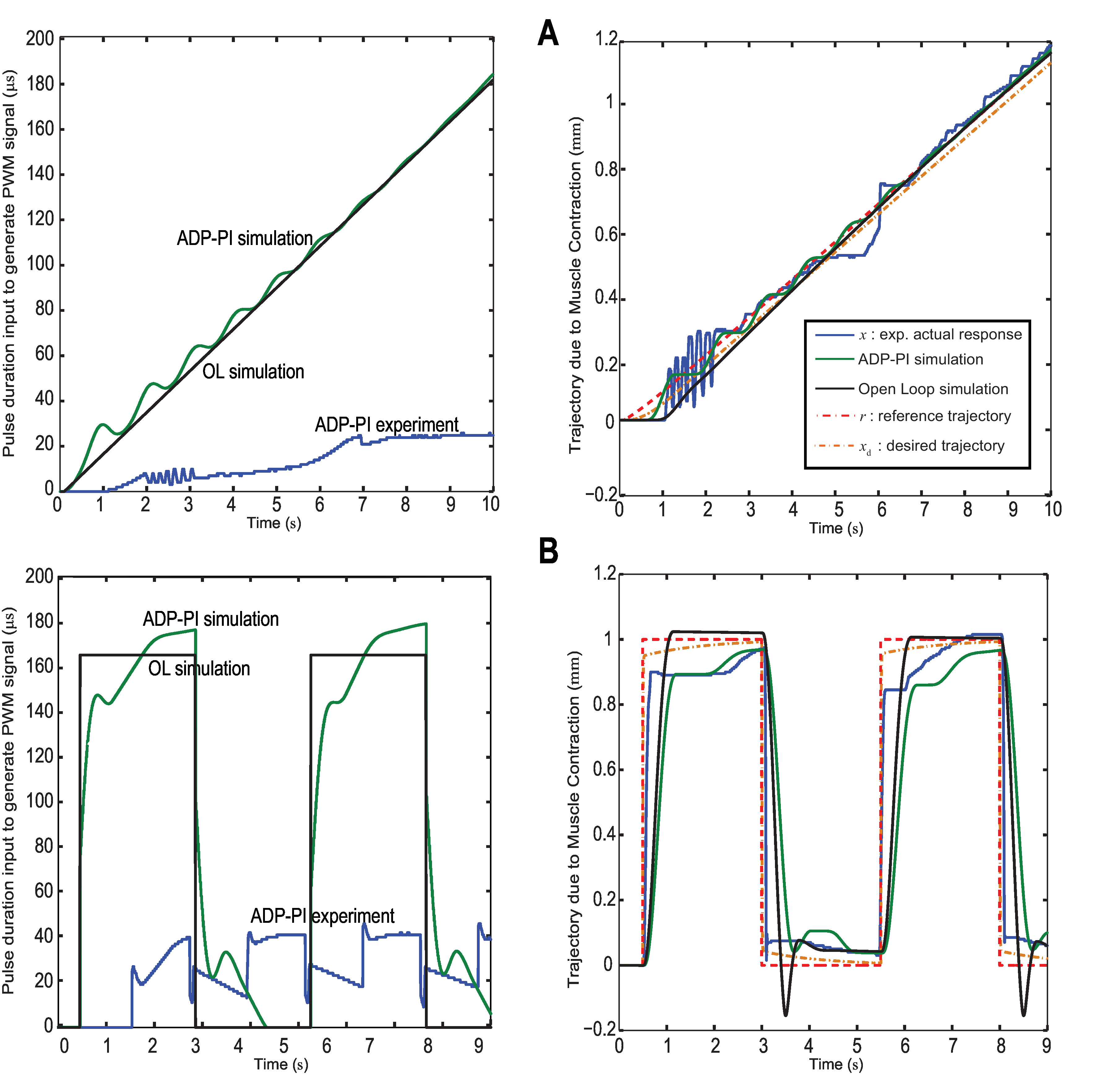

Supplement: S7 Fig — Experimental sample applying the ADP-PI controller for muscle contraction compared to the muscle system simulation for the ADP-PI and open-loop case. Trajectories include (C) ramp and (D) square functions. (TIF) [file pone.0172761.s012.tif]
