# Supplementary material for: Classical and adaptive control of ex vivo skeletal muscle contractions using Functional Electrical Stimulation (FES)
Source: PLoS One. 2017 Mar 8;12(3):e0172761. doi: 10.1371/journal.pone.0172761 (PMC5342220; doi:10.1371/journal.pone.0172761)
Supplement: S3 Table — A typical cascade of events when carrying out an experiment. (PDF) [file pone.0172761.s006.pdf]

S3 Table. Experiment Timeline. Step-by-step guide performing an experiment.

Steps	Time Duration	Procedure
1.	2–4 mins	Caged mouse C57BL10 is euthanized with CO_2.
2.	< 1 min	Mouse is placed in a weigh balance. Mass measurement is recorded.
3.	10–20 mins	Mouse is placed on surgical table to start dissection of the EDL muscle. The muscle is secured with suture at both ends close to the tendons. Only one EDL muscle is used per mouse. Due to euthanization, the other EDL muscle will not be responsive after the duration of the experiment (rigor mortis).
4.	2–3 mins	Isolated EDL muscle is mounted in the mechanical setup, moisten with $(95\%O_2, 5\%CO_2)$ physiological saline solution (PSS). Suture attached to the muscle is secured on both ends by nylon screws. The EDL muscle is submerged and placed parallel to the electrodes.
5.	10 mins	the EDL muscle is set at resting tension; that is 1gF. The optimum length, L_0 is recorded. During this time, the EDL muscle is at rest to acclimate to the PSS prior to the start of the trials. The EDL muscle will remain at resting tension throughout the entire experiment trials.
6.	< 1s	The EDL muscle is subjected to a twitch. At this moment, the muscle is evaluated to determine whether we should continue with the various trials. If the muscle force produced is greater or equal to 5gF, then we start the trials. Force measurements lowered than 5 gF will not generate optimal isometric contractions.
7.	Wait 2-3 mins	Select the step trajectory from the Labview interface. Start tuning the control parameters (PI, MRAC, or ADP-PI) based on the EDL muscle response. Only one controller is used per muscle. Each trial lasts between 9 to 10s. The order of the trajectories is always step, ramp, sine, and square. The control parameters for each trajectory are tuned accordingly. The number of trials per experiment relies on the muscle responses as muscle decay in time.
8.	Rest 30s between trials	Every 5 to 6 trials, the EDL muscle is subjected to a twitch to evaluate the quality of the muscle. After each twitch, the muscle will rest for a duration of 5 mins. The EDL muscle experiments last between 35-50 mins.
9.	After 35-50 mins	The EDL muscle is dismounted. Set on the surgical table to be dried, and for suture removal. Then, the EDL muscle is placed in a weigh balance and the mass measurement is recorded.
10.		Mouse carcass is prepared for disposal per outlined in Virginia Tech IACUC regulations.
